# Construction of a Quantitative Genomic Map, Identification and Expression Analysis of Candidate Genes for Agronomic and Disease-Related Traits in *Brassica napus*

**DOI:** 10.3389/fpls.2022.862363

**Published:** 2022-03-11

**Authors:** Nadia Raboanatahiry, Hongbo Chao, Jianjie He, Huaixin Li, Yongtai Yin, Maoteng Li

**Affiliations:** ^1^Department of Biotechnology, College of Life Science and Technology, Huazhong University of Science and Technology, Wuhan, China; ^2^School of Agricultural Sciences, Zhengzhou University, Zhengzhou, China

**Keywords:** *Brassica napus*, quantitative genomic map, oil content, seed yield, disease, candidate genes, gene expression, structural variation

## Abstract

Rapeseed is the second most important oil crop in the world. Improving seed yield and seed oil content are the two main highlights of the research. Unfortunately, rapeseed development is frequently affected by different diseases. Extensive research has been made through many years to develop elite cultivars with high oil, high yield, and/or disease resistance. Quantitative trait locus (QTL) analysis has been one of the most important strategies in the genetic deciphering of agronomic characteristics. To comprehend the distribution of these QTLs and to uncover the key regions that could simultaneously control multiple traits, 4,555 QTLs that have been identified during the last 25 years were aligned in one unique map, and a quantitative genomic map which involved 128 traits from 79 populations developed in 12 countries was constructed. The present study revealed 517 regions of overlapping QTLs which harbored 2,744 candidate genes and might affect multiple traits, simultaneously. They could be selected to customize super-rapeseed cultivars. The gene ontology and the interaction network of those candidates revealed genes that highly interacted with the other genes and might have a strong influence on them. The expression and structure of these candidate genes were compared in eight rapeseed accessions and revealed genes of similar structures which were expressed differently. The present study enriches our knowledge of rapeseed genome characteristics and diversity, and it also provided indications for rapeseed molecular breeding improvement in the future.

## Introduction

Rapeseed (*Brassica napus*, AACC = 38) is a tetraploid species derived from the natural hybridization between turnip rape (*B. rapa*, AA = 20) and cabbage (*B. oleracea*, CC = 18) (Nagaharu, 1935; Al-Shehbaz et al., 2006; Chalhoub et al., 2014). Both *Brassica* species and the model plant *Arabidopsis thaliana* belong to the *Brassicaceae* family; their separation took place about 14–20 million years ago (Yang et al., 1999; Beilstein et al., 2006). Rapeseed is the second most important oil crop in the world, which could supply 13% of the world’s vegetable oil (Hajduch et al., 2006; Amar et al., 2009).

Rapeseed utilization is not limited to oil sources, it also can be used for food and energy production, remediation, and as sightseeing attraction (Raboanatahiry et al., 2021). To fulfill the global high demand for oil, the main objectives of researchers are to discover ways to increase the oil content and to develop high-yielding varieties, to succeed in sustainable manufacture in the future. Unfortunately, abiotic and biotic factors frequently weaken the rapeseed development, such as the invasion of *Sclerotinia sclerotiorum* (steam rot) and *Plasmodiophora brassicae* (blackleg disease), which resulted in yield losses of 10–80% (Wang Z. et al., 2014) and 20–30% (Wang et al., 2011), respectively, in China. Besides, drought is one of the most devastating abiotic stresses for seed yield, which affects 40% of the worldwide land area (Zhang et al., 2014).

It would be of great interest to find the genetic loci that could control the traits associated with seed yield and quality, and disease traits, simultaneously, for artificial selection breeding. Rapeseed has experienced selection which contributed to the diversification of winter and spring types. The selection also caused region restructuring where genes controlling agronomically important traits are located. Thus, intensive breeding allowed to optimize those important traits such as oil content, flowering time, and pathogen resistance (Chalhoub et al., 2014). Diversity in the same species is because every individual has their uniqueness starting from their genome and it is reflected into their trait characteristics. Diversity among different species could be understood by analyzing their genomes. Variations within the genome are a reflection of breeding events. Genome diversity might be exploited to detect beneficial phenotypes associated with specific loci on the genome and linked to environmental conditions.

Quantitative trait loci (QTLs) are correlated with variations of phenotype and are extensively used for agronomic trait analysis and in plant breeding. Lots of phenotypic traits are usually responsible for the improvement of most crops, they are quantitative in nature, and are influenced both by many genes and environmental conditions (Zhu and Zhao, 2007). QTL mapping could be used to decipher regulatory loci and genetic mechanism of traits (Paran and Zamir, 2003) to identify the genomic regions which are responsible for trait variation, and to establish a link between phenotype and polymorphic markers in random biparental populations (Cao et al., 2010; Jiang et al., 2014). Several research works have revealed that the phenotypic effect of QTLs for one character in one genetic background might produce a different phenotypic effect in another genetic background. For example, *KN* (*KenC-8* × *N53-2*) and *TN* (*Tapidor* × *Ningyou7*) are two populations that were both cultivated in China, the oil content (OC) and detected QTLs were not similar: 41 QTLs for OC were found with a maximum OC of 50.9% in the 202 *TN-DH* lines (Jiang et al., 2014), whereas in the 348 *KN-DH* lines, 63 OC-QTLs were found with maximum OC of 54.8% (Wang et al., 2013), and in 300 *KN-DH* lines, 67 QTLs were detected with a maximum OC of 57.17% (Chao et al., 2017). To uncover the similarities and differences between the discovered QTLs, a consensus map that displays multiple QTLs from different genetic and environmental backgrounds is indispensable.

Earlier, building a consensus map was possible, but it was limited by the difference of markers that were used in different studies (Raman et al., 2013). Now, it can be overcome with a QTL alignment map which has been used for seed oil content and seed yield QTLs by using *B. napus Darmor-bzh* as the reference genome (Liu S. et al., 2016; Chao et al., 2017; Raboanatahiry et al., 2017, 2018). The advantage of QTL alignment is to allow the easy comparison between QTLs and the regions of overlapping QTLs and can be used to uncover the “stable” or “specified” regions for a trait or an environment, but also to detect the pleiotropic loci, i.e., regions that control multiple traits, simultaneously. In our previous studies, regions of overlapping QTLs were displayed: on one hand, the regions involved QTLs of the same traits but originated from different populations (e.g., OC-QTL from *KN* and *TN* which were both cultivated in China and overlapped in the same region), these regions can be qualified as “stable” for Chinese environment, despite the change in populations, or if the QTLs were from two populations which were developed in two different environments (e.g., *KN* in China, and *PT* in Canada), these regions were “stable” for the studied trait (e.g., oil content). On another hand, QTLs of different traits which overlapped in the same region were also found, and they might have a pleiotropic effect for those multiple traits (Raboanatahiry et al., 2017, 2018).

Quantitative trait loci (QTLs) investigation and the discovery of related candidate genes can be done together, this strategy helps to comprehend the authority of these genes over traits (Remington and Purugganan, 2003; Zhu and Zhao, 2007). The identification of candidate genes implies the detection of important genes for agricultural and economic quantitative traits. Candidate genes are present within the QTL regions and are responsible for phenotype variation (Tabor et al., 2002). The effect of these genes on the variation of phenotype could be elucidated *via* investigation on the gene arrangement and the interaction of loci affecting this variation (Zhu and Zhao, 2007). This technique has already been used to identify potential candidate genes in *B. napus Darmor-bzh*, for instance, Chao et al. (2017) used this technique to identify potential candidate genes for seed oil content traits, and found 448 genes underlying 41 oil content QTLs. Moreover, 76 candidate genes were found for 57 QTLs for oil content and fatty acids (Raboanatahiry et al., 2017), and 147 candidate genes were discovered inside a region where 131 yield QTLs were overlapping (Raboanatahiry et al., 2018). Candidate genes can be manipulated to get the most beneficial gene combination to get the maximum profit, especially those genes which were found in the region of overlapping QTLs involving many traits. For instance, LPAT2 and LPAT5 were identified as candidate genes in the QTL interval for oil content in the *KN* population of *B. napus* (Wang et al., 2013; Chao et al., 2017), and the seeds of the mutants *lpat2* and *lpat5* lines displayed a decrease in oil content (Zhang K. et al., 2019).

Additionally, the release of various *B. napus* genome sequences: *Darmor-bzh* (Chalhoub et al., 2014), *Tapidor* (Bayer et al., 2017), *Zhongshuang11—ZS11* (Sun et al., 2017), *Gangan, Shengli, Zheyou7, Quinta, No2127, Westar*, and 1689 other accessions (Song et al., 2021), has represented a precious resource, which will have a tremendous impact on understanding rapeseed accessions diversity, notably the structural variation of regions which are associated with agronomic traits.

In our previous study, regions of overlapping QTLs for a single trait were detected (e.g., oil content or seed yield). In this study, the purpose was to construct a quantitative genomic map and to detect genomic regions that might control multiple traits associated with seed, yield, hormones level, and diseases, simultaneously, and the related candidate genes were also identified. Moreover, the structural variation and gene expression levels of those candidate genes were studied in eight different accessions. Consequently, the ultimate objectives were: (1) to build a quantitative genomic map of QTLs associated with agronomic and disease-related traits to display overlapping regions with multiple traits; (2) to reveal the candidate genes within those regions of overlapping QTLs, to find genes that might have pleiotropic effects on seed composition, seed yield, hormones, and disease, and to analyze their interaction; (3) to identify the homologous of these candidate genes in eight rapeseed accessions and to analyze the genes expression and the structural variation. The present study would enhance the knowledge of rapeseed genome characteristics and diversity, the findings can be used to develop molecular markers associated with the studied traits, and also can provide some guidance for molecular design for breeding. Identified candidate genes might be used to target genomics-based improvement and better seed yield, seed composition, and disease resistance in the future.

## Results

### A Quantitative Genomic Map of Quantitative Trait Locus Controlling Seed Yield, Seed Components, Hormone Level, and Disease-Related Traits

**FIGURE 1:**
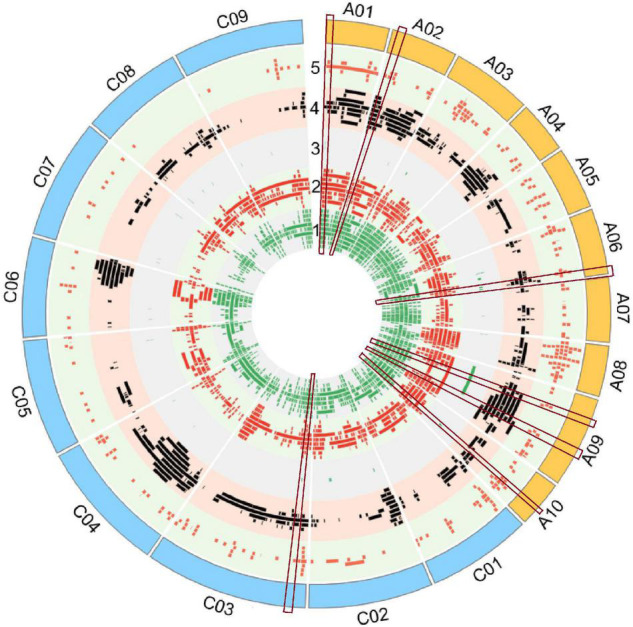
Quantitative trait loci (QTLs) alignment of agronomic and disease-related traits on the physical map of *Darmor-bzh.* QTLs were arranged in each track, from inner to outer circle according to their apparition on the physical map of *Darmor-bzh.* Track 1: Yield-related traits, track 2: Seed composition, track 3: hormone level, track 4: biotic factor, track 5: abiotic factor. The region of overlapping QTLs with five categories of traits is shown inside the red rectangular label. The map was built using Circos software.

**FIGURE 2:**
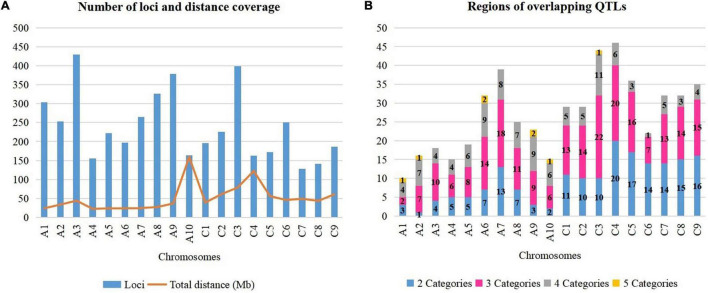
Dissection of rapeseed genome. **(A)** Number of loci and total distance. **(B)** Regions on chromosomes with overlapping QTLs.

A total of 4,555 QTLs of 128 agronomic and disease-related traits, developed from 79 different populations of three different ecotypes and grown in 12 different countries (Supplementary Table 1), were gathered and combined in one unique map (Figure 1). A total distance of 978.4 Mb on the physical map of *Darmor-bzh* was covered (Figure 2A, Supplementary Figure 1, and Supplementary Table 2). Further observation revealed that 2,695 and 1,860 QTLs were located on A and C genomes, respectively. A3 and C3 chromosomes contained the highest number of QTLs, with 430 and 399 QTLs, respectively. A9, A3, and C3 chromosomes contained the highest number of traits (80, 71, and 69 traits, respectively) (Figure 2A). Most QTLs for seed components, seed yield, hormones level, and disease-related traits were found in the A genome rather than in the C genome.

It is crucial to locate regions of the genome where multiple traits overlapped the most. Thereby, the above-mentioned 128 traits were subdivided into five categories: 10 abiotic factor traits (A), nine biotic factor traits (B), four hormones related traits (H), 26 seed components traits (S), and 79 yield-related traits (Y). The total number of QTLs in each category were 349 (A), 334 (B), 42 (H), 1392 (S), and 2,438 (Y). Each region on *Darmor-bzh* genome was carefully observed to detect the regions where QTLs of more than one category of trait could overlap, i.e., regions with two, three, four, or five categories of traits, which were present in one region, simultaneously. A total of 517 regions that hosted overlapping QTLs were observed (Figure 2B and Supplementary Figure 1). The region of overlapping QTLs on each chromosome, the number of QTLs, and the categories of traits are summarized in Supplementary Table 3. First, eight regions were found to harbor all the five categories of studied traits (A, B, H, S, and Y) (Supplementary Table 4). Those eight regions were located on six chromosomes: one region was found on each of A1 (1.71–1.71 Mb, with 40 QTLs), A2 (2.31–2.31 Mb, with 20 QTLs), A10 (11.78–11.87 Mb, with 14 QTLs), and C3 (5.09–5.33 Mb, with 11 QTLs), and two regions were found on A6 (21.68–21.95 Mb with 15 QTLs and 22–22.30 Mb with 13 QTLs), and A9 (8.12–9.87 and 20.76–22.51 Mb, with 34 QTLs on each of them). Second, 107 regions that contained four categories of traits were found in all 19 chromosomes. The number of regions in each chromosome was, respectively, 11 on C3, nine on each of A6 and A9, eight on A7, seven on each of A2 and A8, six on each of A6, A10, and C4, five on C1 and C2, four on each of A1, A3, A4, and C9, three on each of C5 and C8, and one on C6. For example, 28 QTLs of four categories of traits (1A, 12B, 5S, 10Y) overlapped on A2 (1.49–2.31 Mb). Note that the region on A2 (1.71–22.04 Mb) included 288 overlapping QTLs (12A, 22B, 63S, and 191Y), which was the richest region of overlapping QTLs in *B. napus* genome. Third, 225 regions on all 19 chromosomes were found to have overlapping QTLs involving three categories of traits: 22 on C3, 20 on C4, 18 on A7, 16 on C5, 15 on C9, 14 on each of A6, C2, and C8, 13 on each of C1 and C7, 11 on A8, 10 on A3, nine on A9, eight on A5, seven on each of A2 and C6, six on each of A4 and A10, and two on A1. For instance, on a region of A5 (3.49–5.29 Mb), 40 QTLs of three categories of traits (5B, 12S, 23Y) overlapped. Fourth, 177 regions were found to contain overlapping QTLs which involved two categories of traits: 20 on C4, 17 on C5, 16 on C9, 15 on C8, 14 on each of C6 and C7, 13 on A7, 11 on C1, 10 on each of C2 and C3, seven on each of A6 and A8, five on each of A4 and A5, four on A3, three on A1 and A9, two on A10, and one on A2. As an example, 13 QTLs of two categories of traits (10B, 3Y) overlapped on C4 (20.66–20.70 Mb).

Note that some QTLs might overlap multiple times with other QTLs in different regions because of their extended length, for example, a QTL for C16:0 was located on A1 (2.25–19.86 Mb) and it could overlap two times with QTLs in the region which involved five and four categories (1.71–1.71 and 1.71–22.04 Mb, respectively). Then, the most abundant and the most overlapping categories of traits were S and Y categories, they were found in 403 among the 517 regions of overlapping QTLs detected in this study. OC and seed yield (SY) are the two most important traits in rapeseed, and the seed yield is determined by the number of seeds per siliques (SNP), the number of siliques per plant, the seed weight (Qzer et al., 1999; Quarrie et al., 2006; Chen et al., 2007), and the overlapping QTLs for OC and SY traits were observed in 82 regions (Supplementary Table 5), where the chromosome A9 had the richest regions with 11 regions of overlapping QTLs for OC and SY. The H category of the trait was found rarely in the overlapping region since the identified QTLs in early published papers were few (42 QTLs), so far, this H category was found in 39 among the 517 regions of the overlapping QTLs of this study. Otherwise, the regions of overlapping QTLs which involved one environment or one population were observed. No specified region was found exclusively for one population. Also, only specified regions of overlapping QTLs, which involved some populations developed exclusively in China, were found in 11 areas of the genome: four areas on C3 (36.94–37.27, 37.27–38.94, 39.94–40.21, and 41.40–46.52 Mb), two areas on A8 (16.87–17.37 and 17.37–18.00 Mb), and one area on each of A7 (17.48–18.48 Mb), A10 (0.14–1.64 Mb), C4 (42.73–44.22 Mb), C6 (8.43–9.43 Mb), and C7 (24.87–25.45 Mb) (Supplementary Table 3). For instance, the region on A8 (16.87–17.37 Mb) had four QTLs (3S, 1Y), which were all found with the Chinese experimental field. Besides, “hot QTL regions” had been detected in four locations on the rapeseed genome. Those regions were enriched with more than 100 QTLs which were aligned: on chromosome A1 (1.71–22.04 Mb), a total of 228 QTLs of four categories of traits (A, B, S, Y) was found. Chromosome A3 (0.63–6.75 Mb) had 139 QTLs of three categories of traits (B, S, Y), and A8 (9.63–11.12 Mb) contained 111 QTLs of four categories of traits (A, B, S, Y). Then, chromosome C6 (29.29–36.52 Mb) was enriched with 144 QTLs of three categories of traits (B, S, Y). Ultimately, the rapeseed genome had been finely dissected to unveil regions that harbored multiple traits, simultaneously. It would be crucial to couple those findings with the identification of genes that were located within those regions to understand the influence of those genes over those traits.

### Candidate Genes Identified Within Regions of Overlapping Quantitative Trait Locus, and Their Interaction Network

**FIGURE 3:**
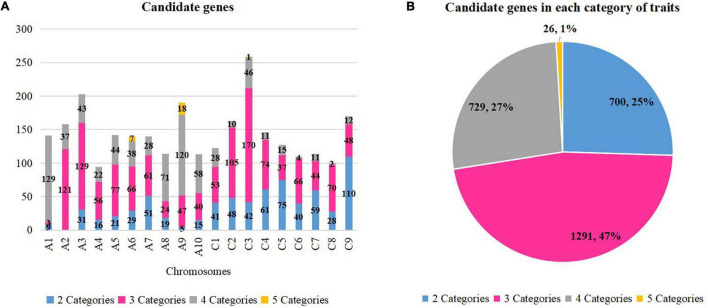
Candidate genes in each category of traits. **(A)** Candidate genes in each chromosome. **(B)** Candidate genes in each category of traits. The number and percentage of genes in each category are displayed. Different colors correspond to different categories.

Previous studies identified 439 genes that were related to oil formation (Raboanatahiry et al., 2017), 1,398 genes that were related to yield traits (Raboanatahiry et al., 2018), and 1,344 genes which were resistance genes (Dolatabadian et al., 2017). Thus, a total of 3,181 genes were selected because they correlated with the studied traits, and they were aligned to the physical map of *Darmor-bzh* to detect the candidate genes. A total of 2,744 candidate genes were found within overlapping QTLs of two to five categories of traits (Figure 3A and Supplementary Table 6). A total number of genes of 26 (1%), 729 (47%), 1,291 (27%), and 700 (25%) were found for five, four, three, and two categories of traits, respectively (Figure 3B).

Eight regions of the overlapping QTLs of five categories of traits (A, B, H, S, and Y) were found in six chromosomes (A1, A2, A6, A9, A10, and C3). A total of 26 candidate genes were found on three among those six chromosomes: seven genes on A6 (four on 21.68–21.95 Mb and three on 22–22.30 Mb), 18 genes on A9 (six on 8.12–9.87 Mb and 12 on 20.76–22.51 Mb), and one gene on C3 (5.09–5.33 Mb). For example, three candidates were found on A6 (22–22.30 Mb) which were a *DHLAT* gene (BnaA06g33300D), an *RLK* gene (BnaA06g33320D), and an *AAPPT* gene (BnaA06g33540D). Meanwhile, 729 candidate genes were found within overlapping QTLs of four categories of traits in all 19 chromosomes, and they were, respectively, of 129 (A1), 120 (A9), 71 (A8), 58 (A10), 46 (C3), 44 (A5), 43 (A3), 38 (A6), 37 (A2), 28 (A7 and C1, each), 22 (A4), 15 (C5), 12 (C9), 11 (C4 and C7, each), 10 (C2), four (C6), and two (C8). For example, three candidate genes (*CCT* BnaA03g14860D, *RLK* BnaA03g15210D, and *KAT2* BnaA03g15290D) on A3 (6.84–7.12 Mb) were found within 19 overlapping QTLs (2B, 1H, 5S, 11Y). Moreover, 1,289 candidate genes were located within overlapping QTLs of three categories of traits, and they were found on all 19 chromosomes: 169 (C3), 129 (A3), 121 (A2), 104 (C2), 77 (A5), 74 (C4), 70 (C8), 66 (A6 and C6), 61 (A7), 56 (A4), 53 (C1), 48 (C9), 47 (A9), 44 (C7), 40 (A10), 37 (C5), 24 (A8), and three (A1). For instance, two candidate genes (*ADC2* BnaC01g03710D, and *FAE* BnaC01g04130D) were found on C1 (1.93–2.16 Mb) involving 13 overlapping QTLs (4B, 1S, 8Y). At last, overlapping QTLs of 2 categories of traits contained 700 candidate genes in 18 chromosomes (excluding A2): 110 (C9), 75 (C5), 61 (C4), 59 (C7), 51 (A7), 48 (C2), 42 (C3), 41 (C1), 40 (C6), 31 (A3), 29 (A6), 28 (C8), 21 (A5), 19 (A8), 16 (A4), 15 (A10), 9 (A1), and 5 (A9). For example, two candidate genes (*RLK* BnaC07g13860D and *RN* BnaC07g14020D) were found on C7 (19.60–19.79 Mb) involving two overlapping QTLs (1 A and 1 B). In assumption from those findings, important genes which were located within regions of overlapping QTL with multiple traits were identified. They might influence more than one category of traits, and they could be selected according to the desired improvement of two or multiple traits.

The interaction network analysis of the 2,744 candidate genes was made with their 1,555 orthologous genes in *A. thaliana* because *B. napus* are not available on the String database. Gene ontology (GO) analysis indicated that the 1,555 genes could be classified into 16 categories, according to Panther GO-slim biological process’s classification (Supplementary Table 7): it included the cellular process, biological phase, reproductive process, multi-organism process, localization, interspecies interaction between organisms, reproduction, biological regulation, response to stimulus, signaling, developmental process, rhythmic process, multicellular organismal process, metabolic process, growth, immune system process. Other genes which could not fit into those categories were classified as “Others.”

**FIGURE 4:**
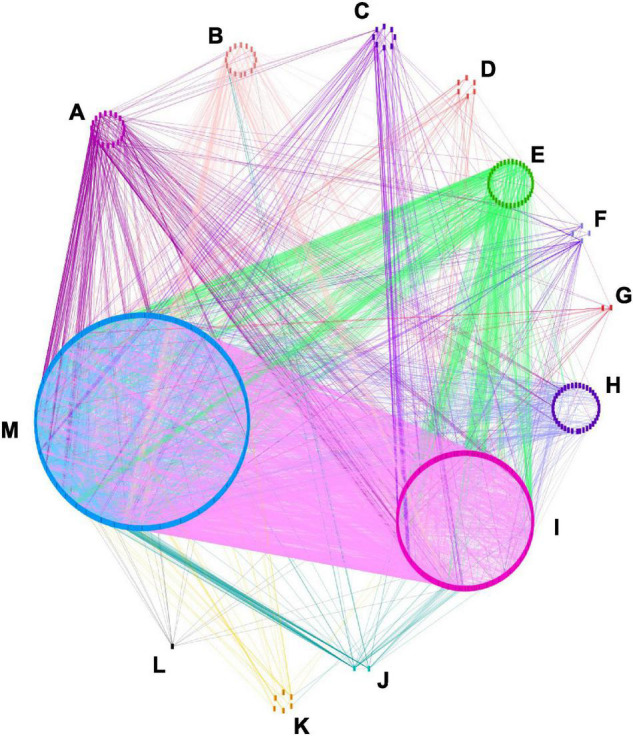
Candidate genes interaction network. The interaction analysis was made with orthologous *A. thaliana* genes by using STRING (http://string-db.org/) and visualized with Cytoscape_V3.8.2. 1271 nodes and 10101 edges are shown. Eleven categories of genes are displayed according to their GO term enrichment. **(A)** Signaling, **(B)** multicellular organism, **(C)** growth, **(D)** biological regulation, **(E)** cellular process, **(F)** developmental process, **(G)** immune system process, **(H)** localization, **(I)** metabolic process, **(J)** rythmic process, **(K)** response to stimulus, **(L)** reproduction, **(M)** others.

The interaction network was visualized with Cytoscape and 1,271 nodes and 10,101 edges were displayed (Figure 4). In this network, 34 genes might be more influential over other genes (degree layout, DL≧60) (Supplementary Table 8). Those genes belonged to the GO categories of the cellular process, metabolic process, multicellular organismal process, rhythmic process, and “others” category. *ACP, DGAT, KASI, KASII, KASIII, LPAAT*, and *MCMT* had functions related to oil biosynthesis (Li-Beisson et al., 2013), *AGL20, AP2, AUX1, CO, COP1, EMB, FLC, FLD, FRI, FT, FVE, GI, LPAAT, PHYA, PHYB, RGA, SVP, TFL1*, and *TFL2* were related to yield traits, while A*P40, ARP, ERD, GMP synthase, HEME, SH3*, and *WUS* were involved in plant resistance to disease (Poole, 2007). The 26 candidate genes detected in the region of overlapping QTLs involving five categories of traits had less influence over the other genes DL < 46 (Supplementary Table 9), in comparison to the 11 above-mentioned genes. The most influential genes had different functions and were involved in different metabolism pathways, yet they might have a higher effect over other genes, this might indicate that the simultaneous control of multiple categories of traits might be affected at different paths of metabolisms.

### Gene Expression and Structural Variation of Candidate Genes in Eight Rapeseed Accessions

**FIGURE 5:**
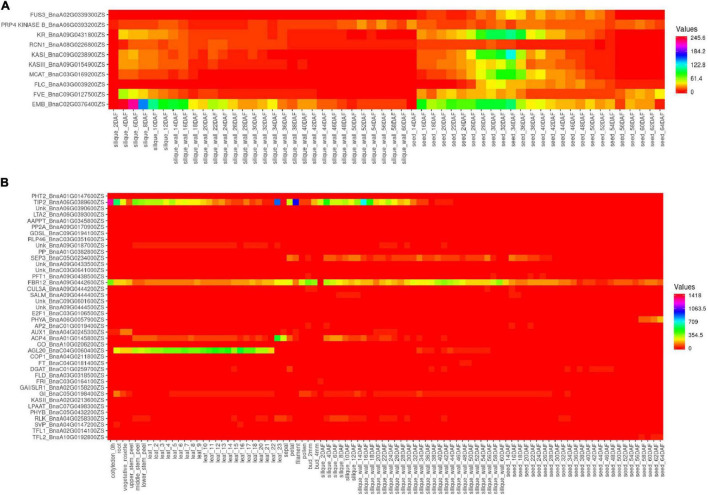
Expression level of the candidate genes in *ZS11.* **(A)** Ten genes exclusively with higher expression in the siliques and seeds. **(B)** Expression of other genes in different tissues. The heatmap was built using Heatmapper (http://www2.heatmapper.ca/).

The 34 most influential genes of the interaction network in *B. napus* were selected for the current gene expression analysis. Additionally, the 26 candidate genes which were found within QTLs of five categories (A, B, H, S, and Y) were also added to the analysis (Supplementary Table 10). Two groups of genes could be observed, the first group was the genes that were mainly expressed in the seeds and siliques, including *FUS3, PRP4 KINASE B, KR, RCN1, KASI, KASIII, MCAT, FLC, FVE*, and *EMB* (Figure 5A and Supplementary Table 11), with higher expression in the seeds rather than in the siliques, except for the *EMB* gene. The second group had the genes expressed in different tissues and the genes with very low expression levels (Figure 5B and Supplementary Table 11).

**FIGURE 6:**
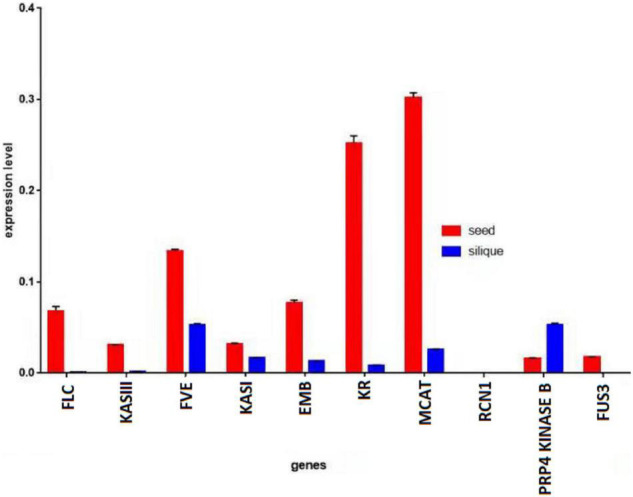
Expression level of genes in seeds vs. siliques of *KenC-8. Actin* was used as reference gene.

A quantitative PCR (qPCR) was performed using the complementary DNA (cDNA) from the seeds and siliques of *KenC-8.* It was observed that the genes had higher expression in the seeds than in the siliques, except for the *PRP4 KINASE B* gene which had higher expression in the siliques than in the seeds. Moreover, the *EMB* gene which had a higher expression in the siliques than in the seeds of *ZS11*, had a higher expression in the seeds than in the siliques of *KenC-8* (Figure 6).

**FIGURE 7:**
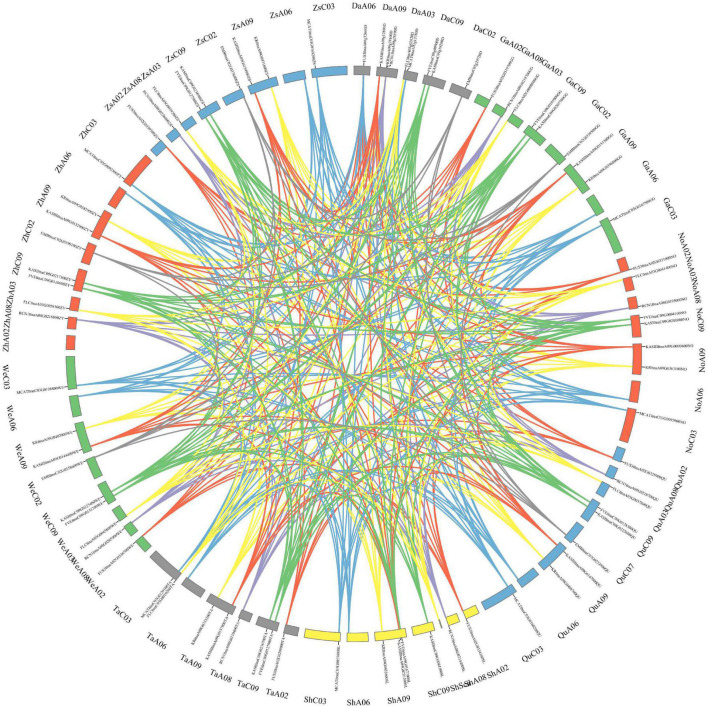
Synteny of the ten genes in nine rapeseed varieties. The map was built with TBtools software, with *Darmor-bzh (Da), Gangan (Ga), No2127 (No), Quinta (Qu), Shengli (Sh), Tapidor (Ta), Westar (We), Zheyou (Zh), Zhongshuang 11 (Zs).* Chromosome location is displayed near the genome name.

Besides, the gene expression and the structural variation of the above-mentioned 10 genes were analyzed in eight rapeseed varieties, including two winter-types (*Quinta* and *Tapidor*), two spring-types (*Westar* and *No2127*), and four semi-winter types (*ZS11, Zheyou7, Shengli* and *Gangan*). The synteny of the genes in the nine rapeseed varieties is shown in Figure 7. The genes were located in five chromosomes in *Darmor-bzh*, and in eight chromosomes in the other eight rapeseed varieties. The length of the genes was almost similar in the eight varieties but they were much longer in size in *Darmor-bzh* (Supplementary Table 12). The expression of those 10 genes was compared in the eight rapeseed varieties (Supplementary Figure 2). The nucleotide sequence identity was analyzed (Supplementary Table 13). Genes with a higher rate of sequences identity had similar gene expressions. It was the case of the *KASIII* genes in *Zheyou* and *ZS11.* However, genes that displayed 100% of sequence identity could also have different expression profiles, such as the *KR* genes of the eight varieties that shared 100% of sequence identity but displayed a different expression level. More, some other genes with different sequences identity showed similar expression profiles, like the case of *FLC* genes in *Gangan* and *No2127.*

## Discussion

The current study aimed to combine QTLs for seed component, seed yield, hormones, and disease-related traits, which were detected in previous studies, in one physical map in rapeseed, to identify the related candidate genes and to analyze their expression and structural variation in different rapeseed accessions. The same strategy was used to find the regions that might control multiple traits of one category, i.e., seed oil (Raboanatahiry et al., 2017) and seed yield (Raboanatahiry et al., 2018). In those two studies, some regions were suggested to possibly contribute to the improvement of one trait or multiple traits of one category, and some regions were supposed to be stable for one given environment. For example, a region on A1 (2.50–2.99 Mb) had overlapping QTLs for plant height, which was from two populations developed in China (*Tapidor* × *Ningyou7* and *Express617* × *V8*), so this region might affect the plant height and it is a stable region for the Chinese environment (Raboanatahiry et al., 2018). More, QTLs for C16:0, C18:0, C18:1, C18:2, C20:0, and C22:1 were overlapping on C3 (53.75–58.29 Mb), thus, this region might control those six traits, simultaneously (Raboanatahiry et al., 2017). The current study was made at a higher level because it was not limited to one category of trait as in the previous studies, but five categories which involved almost all the studied traits in rapeseed.

Increasing seed oil and seed yield is among the main focus of researchers on rapeseed, to cater to the increasing demand for

oil. However, the usage of rapeseed is not limited to biomass for oil, but also multiple purposes, such as protein, carbohydrate, vitamins sources, and many more (Raboanatahiry et al., 2021). Despite the effort in improving seed components and seed yield, rapeseed crops are under attack from various diseases that resulted in huge crop loss. For example, *Leptosphaeria maculans* causes blackleg disease (West et al., 2001), which has created an economic loss of $900 million per growing season globally (Fitt et al., 2008). Although resistant cultivars have been developed and cultivated since the 1990s in Canada (Kutcher et al., 2010), which has decreased the yield loss by 1% (Gugel and Petrie, 1992), abiotic stresses have also caused about 50% yield reduction in major crops (Bray et al., 2000). Extensive research is still undertaken to take total control of those biotic and abiotic diseases, *via* selective breeding. Otherwise, phytohormones play important roles in plant growth and development, such as IAA (Grossmann, 2010), but also on plant adaptation to assure survival to face the environmental fluctuation. Abscisic acid (ABA) responds to both biotic and abiotic stresses (Cao et al., 2011), which have an influence on one another (Fujita et al., 2006). Those phytohormones support agronomic trait improvement and response to disease. Therefore, all the five categories of traits analyzed in the current study are correlated and are pivotal for rapeseed crop improvement.

### Dissection of Rapeseed Genome Revealed Regions Controlling Multiple Traits

The current study is the first study to gather all the QTLs of important agronomic and disease-related traits discovered in *B. napus* over 25 years, to construct a quantitative genomic map, which is crucial to uncover similarities and differences in QTLs detected from different populations and environments, but also to reveal the regions that might control multiple beneficial traits, simultaneously.

It was obvious that most of the QTLs were found on the A rather than C genome (2,695 vs. 1,860 QTLs, respectively). Selection has played important role in improving *B. napus*. It was reported that the C genome rather than the A genome, contained extended breeding regions (51.15 Mb on C vs. 16.80 Mb on A) which might contribute more to alleles producing elite traits (Wang Z. et al., 2014). However, a recent investigation on the origin of *B. napus* and the genetic loci that contributed to its improvement had revealed that the parallel selection of the A and C genomes had led to seed quality improvement in *B. napus* (Lu et al., 2019). “A” genome-specific selection greatly enhanced disease resistance, oil accumulation, and environment adaptation of *B. napus* during its first stage of improvement, while the C genome had improved developmental traits. This might explain the fact that most of the QTLs of studied traits in the current study could be found on the A genome. Particularly, for Asian *B. napus* varieties, it was reported that they have experienced strong artificial selection from the A genome which contributed to their adaptation following their introduction from Europe (Zou et al., 2019).

Apart from that, 517 regions were found with overlapping QTLs involving at least two categories of traits. Those regions might be suitable for selection to improve two or more desired traits, simultaneously, for example, to improve both abiotic stress response, seed component, and seed yield (A4:3.07–4.11 Mb). Several studies have already investigated on the co-location of QTLs from a different category of traits (Shi et al., 2012; Zhao J. et al., 2012; Ding et al., 2014; Bouchet et al., 2016; Körber et al., 2016; Stein et al., 2017; Wan et al., 2017; Rahaman et al., 2018; Jan et al., 2019; Wrucke et al., 2019; Wu et al., 2019; Zhang K. et al., 2019). Those studies demonstrated the importance of analyzing multiple traits, at the same time, to target the loci for breeding cultivars with the most advantageous profile. For example, a study that focused on flowering time (FT) and *Sclerotinia* stem rot resistance (SSR) reported that early FT might increase susceptibility to *S. sclerotiorum*, and regions of co-location of FT and SSR resistance traits were found which were crucial for breeding early maturing and SSR resistance cultivars. Moreover, four co-localized QTL hotspots of SSR resistance and FT on A2 (0–7.7 Mb), A3 (0.8–7.5 Mb), C2 (–15.2 Mb), C6 (20.2–36.6 Mb), which were consensual with previous studies (Wu et al., 2019). In the current study, the QTL of SSR and DIF (FT) were also co-localized in those regions.

Particularly, seed components and seed yield traits often overlapped in this study. In earlier studies, yield traits such as the flowering time, the morphology of the root, and the plant growth environment could affect seed quality traits such as erucic acid, oil, protein, and glucosinolate contents (Paran and Zamir, 2003; Si et al., 2003; Quijada et al., 2006; Shi et al., 2013; Wang X. et al., 2017). In oil crops, QTLs that could influence both seed quality and yield traits had already been discovered in several studies, positively or negatively. For instance, oil and protein contents were positively correlated with seed weight in 11 *B. carinata* lines developed in Canada (Getinet et al., 1996). Zhao et al. (2006) found evidence of a positive correlation between oil content and seeds per silique while evaluating 282 *DH* lines from a cross between *Sollux* and *Gaoyou* (*B. napus*), and developed in Germany and China. In a study performed by Chen et al. (2010) in a *DH* population derived from a cross between high and low oil content *B. napus* and developed in Canada, oil content and flowering time were negatively correlated. In that study, QTLs for oil content, flowering time, and seed yield were co-localized on a small region of LG7 where alleles of low oil content, early flowering time, and higher seed yield were found together. However, QTLs for high oil content and early flowering time were found in the co-location on LG2. Otherwise, since oil content and seed yield are the most important trait in rapeseed, 82 QTL regions were discovered, where QTLs for these two traits overlapped. They could be selected to improve the two traits, simultaneously.

Overlapping QTLs of multiple traits might happen when gene alteration frequencies at nearly linked loci occur, but also, it might be caused by the pleiotropic effect when an appropriate substitution of genes occurs (Smith and Haigh, 1974). Also, pleiotropy or/and linked genes might have caused this phenomenon. Hot QTLs regions were also discovered with more than 100 aligned QTLs, which is interesting for rapeseed molecular breeding, because the region with the QTLs of diverse traits could be targeted to improve multiple traits, simultaneously, and the more the number of QTLs increased in that region, the more the locus was stable because, despite the diversity in population and environment background, it did not alter the location of the QTL on the genome.

The region on the chromosome which was exclusively for QTLs from one population was not found, and those exclusively for one environment were for China only (fixed environment for China). This indicated that those QTLs remained unchanged, despite the variation of population and environment.

### Identified Candidates Genes Might Be Pleiotropic or Linked Genes?

In previous studies, 110 genes in *Arabidopsis* were identified to be involved in oil formation (Li-Beisson et al., 2013) and 439 homologous genes were found in *B. napus* (Raboanatahiry et al., 2017). Moreover, 425 yield genes which were related to branch number, flowering time, maturity time, plant height, pod number, seed number, seed weight, and seed yield in *Arabidopsis* were identified (Shi et al., 2009) and 1,398 homologous genes were detected in *B. napus* (Raboanatahiry et al., 2018). Dolatabadian et al. (2017) found 1,344 resistance genes in *B. napus*. Those genes had a relationship with the currently studied traits, thus, they were selected to uncover the candidate genes.

The function of those selected genes correlated with the studied traits. Since many genes could be found within a region of a genome, a preselection is necessary, so that the candidates have a close connection to the studied traits, and have a functional outcome on the process (Zhu and Zhao, 2007). A total of 2,744 genes related to oil, yield, and resistance were found within overlapping QTLs involving two to five categories of traits. As mentioned above, overlapping QTLs might be caused by pleiotropy or linked genes. Pleiotropy is when one gene can control multiple unrelated phenotypic traits (Stearns, 2010; Conner et al., 2011; Wagner and Zhang, 2011; Solovieff et al., 2013). Pleiotropy is largely distributed due to biochemical and developmental systems and it affects development and evolution and creates correlations between genes and phenotype, and it affects selection and imposes the accessibility of the evolution extent (Gromko, 1987; Lynch and Walsh, 1998). The pleiotropic organization of traits (dominant or epistatic) can be modified by selection and inbreeding (Goodnight, 1988; Cheverud and Routman, 1996; Hansen and Wagner, 2001). Linked genes are genes located close to each other on the same chromosome and are inherited together during meiosis. Genes might separately control different phenotypes but are found closely located on the same region of a chromosome.

**FIGURE 8:**
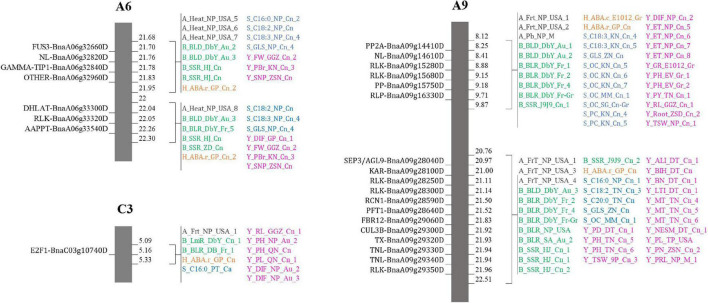
Suggestions for selective breeding. Regions on A6, A9, and C3 chromosomes where QTLs of five categories overlapped.

Candidates found within the region of overlapping QTLs with five categories of traits attracted more attention since they might be more influential than the others over multiple traits. A total of 26 candidates were found on five regions distributed on A6, A9, and C3 chromosomes, and they would be the most recommended in this study, for genomic selection to target multiple traits simultaneously (Figure 8). They belonged to different families and might have different distinct roles, but the way they act to influence each other or to affect the studied traits still needs a deep investigation. Functional investigation of each gene over the studied traits would be indispensable to comprehend their influence on the traits and would reveal whether they were pleiotropic genes or linked genes.

Gene interaction network revealed that 34 genes might have more influence over the other genes. Genes are responsible for the genetic variation of traits (Tabor et al., 2002), and the structure and dynamism of the genetic regulatory network have an impact on quantitative traits (Frank, 2003). In this study, *KAS, ACP, AUX1, CO, FT, PHYA, and AGL20* were also identified as the most influential genes in our previous studies (Raboanatahiry et al., 2017, 2018). Despite the number of genes identified in this study being far larger than those of the previous study, and the gene function were also broader, those seven genes of different functions still had higher influence over the other genes, indicating that simultaneous control of multiple traits might be affected at different metabolism pathways.

The expression analysis of the candidate genes revealed an exclusive increase for 10 genes (*FUS3, PRP4 KINASE B, KR, RCN1, KASI, KASIII, MCAT, FLC, FVE*, and *EMB)* in the seeds and siliques of *ZS11*, with higher expression in the seeds than in the siliques, except for *EMB.* Reversely, the *PRP4 KINASE B* of *KenC-8* had increased expression in the siliques in comparison to the seeds. Previous studies reported the function of those 10 genes: *MCAT, KASI*, and *KASIII* (β*-ketoacyl-ACP synthase I* and *III*), and *KR* (β*-ketoacyl- ACP reductase)* are enzymes of fatty acid biosynthesis. *MCAT (Malonyl-CoA:ACP transacylase)* was suggested to be essential for a high oil content *B. napus*, and might be used to improve the seed oil contents (Qu et al., 2014). Plastidial *KASIII* genes could alter the fatty acid profile of *B. napus* seeds, by increasing the C18:2 and C18:3 contents at the expense of C18:1 (Verwoert et al., 1995). *KASI* played a positive role in *Arabidopsis* morphology and fertility, and in polar lipid composition. Moreover, *KASI* disruption affected embryo development and decreased the fatty acid contents (Wu and Xue, 2010). *FUS3 (FUSCA3)* is a regulator of seed development and seed oil content. It induces the genes of fatty acid biosynthesis during development in *Arabidopsis* (Wang et al., 2007). Likewise, the oil production decreased in *fus3* of *B. napus* (Elahi et al., 2015). In Arabidopsis, *RCN1 (roots curl in naphthylphthalamic acid1)* encodes a regulatory α-subunit of protein phosphatase 2A. *RCN1* modulates auxin responses in roots (Garbers et al., 1996), and defection resulted in increased basipetal auxin transport and a significant delay in gravitropism (Rashotte et al., 2001). Moreover, *rcn1* roots had a reduced elongation in seedlings and hypocotyl elongation (Muday et al., 2006). *FLC (FLOWERING LOCUS C)* is a vernalization regulator with a high expression level in winter-type rapeseeds (Schiessl et al., 2019). It induces a delayed flowering time in *Arabidopsis* and *Brassica rapa* (Kim et al., 2007), but also in *B. napus* (Tadege et al., 2001). However, *FLC* could be repressed by *FVE* genes which act on the regulation of flowering time (Baek et al., 2008; Yu et al., 2016). *EMB (EMBRYO DEFECTIVE) is* required for growth and development in *Arabidopsis* (Devic, 2008; Meinke, 2020). In the current study, the particular presence of those genes in the seeds indicated their importance at an early stage of plant formation.

The expression and structural variation analysis of the genes in eight rapeseed varieties showed that some genes which had 100% sequence identity displayed different expression profiles, and some other genes with different sequences identity showed similar expression profiles. Note that Vector NTI software (Invitrogen) was used to calculate the sequence identity, and it was observed that even with a large structural variation, the software still displayed a 100% of sequence identity between the genes, which was unexpected. However, genes with several SNPs had less than 100% of sequence identity. Several SNPs (case of *KASI*) and a large structure variation (case of *MCAT* genes) in the candidate genes were observed, which might explain the difference in expression. This probably implied that trait variations possibly occur because of the structure and expression variation of the candidate genes, which can be verified in future studies.

Epigenetics is one of the factors which might cause an alteration in gene expression while preserving the primary DNA sequence or genotype (Bird, 2002; Tchurikov, 2005). Epigenetic mechanisms include DNA methylation which commonly induces gene silencing by blocking the transcription binding sites, histone modification which alters chromatin structure and accessibility of genes for transcription, and non-coding RNA-associated gene silencing which targets mRNA transcripts for destruction induce and preserve epigenetic change (Egger et al., 2004; Tirado, 2014). Even if an epigenetic change is natural and regular, it can also be influenced by environmental factors (Aguilera et al., 2010; Feil and Fraga, 2012). In the case of our study, further analysis is needed to conclude about epigenetic *via* comparison of promoter sequence between genes. Because the full genomic sequences are absent in Brassica napus pan-genome information resource (BnPIR), the analysis could not be done in the current study. Also, the eight accessions were produced with different genetic and environmental backgrounds, and the age of plant materials also plays a role in gene expression. Thus, the difference in the gene expression even with a similar sequence was expected.

### Breeding a Super-Rapeseed Cultivar That Meets Expectations

The current study uncovered regions, with two, three, four, or five categories of traits that can be chosen and used for marker-assisted selection, to produce a customized rapeseed cultivar with desired traits. For instance, stresses imposed by heat are detrimental to seed yield and quality (reviewed by Sehgal et al., 2018). To control these traits at once, the region on A3 (11.40–12.47 Mb) could be selected for fine-mapping, since it contained overlapping QTLs for heat, seed yield, and seed composition. Candidate genes included in this region could be cloned and validated through functional analysis, to understand the related molecular mechanism.

Another innovation of the current study is the usage of the rapeseed pan-genome of BnPIR to compare the gene expression and gene structure of candidate genes. This strategy aimed to comprehend how the same genes of different accessions would be expressed, and how their structures are different. This might serve later to explain their functions. Since numerous rapeseed accessions have been sequenced, performing the same study as our current study is now feasible in those other accessions. It would enhance our understanding of rapeseed genome variation. In the future, it would be interesting to know whether the QTLs of multiple traits could also be found overlapping in the same region of the other rapeseed varieties, as found in *Darmor-bzh* of this study. It is important to discover if the regions were maintained in all the varieties of rapeseed. The characterization of haplotypes is also needed to understand if those regions could be inherited together.

Besides, compared to rice, the rapeseed breeding program needs more effort and innovation. Until now, rapeseed research focused on QTLs and the studied traits were repetitive. However, the rice breeding program already focuses on QTG (quantitative trait gene) and QTN (quantitative trait nucleotide) for the improvement of this crop (Wei et al., 2021). This effort was made to further close the gap between genomic studies and practical breeding, and to facilitate the localization of causative variants of all known traits. A collection of rice varieties with those variations was made and a genome navigation system was established for breeding. Thus, research on rapeseed should switch progressively into those QTG and QTN analyses. Multiple rapeseed accessions are also available and a collection of variations should be implanted for breeding.

Finally, the current study has enhanced our knowledge of rapeseed genome characteristics and diversity. Co-localized QTLs might have an ally or antagonistic effect. For the usage in practical breeding, identification of the most favorable alleles combinations which will produce maximum profits is still crucial.

## Materials and Methods

### Alignment of Quantitative Trait Locus on the Physical Map of *Darmor-bzh*

Extensive literature inquiry allowed us to identify more than 350 papers that reported on genome-wide association studies (GWAS) and QTLs analyses in *B. napus* over the last 25 years (1995–2020). They were manually sorted according to data availability. Research articles with missing information were removed (absence of flanking markers, marker sequence, or physical position of QTLs on *Darmor-bzh*). QTLs/GWAS with just one flanking marker were kept and given an area of 1 cm from the unique marker as loci. A total of 4,555 QTLs for seed, yield, hormones, and disease-related traits were collected from 145 research articles, involving 79 different populations of three different ecotypes and grown in 12 different countries (Supplementary Table 1). They were aligned in the physical map of *Darmor-bzh*. The location of QTLs flanking markers on the physical map was detected via e-PCR (Schuler, 1997; Rotmistrovsky et al., 2004), and the method of alignment was as similar as our previous studies (Raboanatahiry et al., 2017, 2018). The map was built using Circos software (Krzywinski et al., 2009).

### Identification of Candidate Genes

Genes in *B. napus* which were reported in three different pieces of literature were selected as a basis for the identification of candidate genes in the current study: 439 genes were related to oil formation (Raboanatahiry et al., 2017), 1,398 genes were related to yield traits (Raboanatahiry et al., 2018), and 1,344 genes were resistance genes (Dolatabadian et al., 2017). They were aligned into the physical map of *Darmor-bzh*, and the genes located within overlapping QTLs were identified as candidates for the traits.

### Construction of Gene Interaction Network

The gene interaction network was predicted using STRING (Szklarczyk et al., 2015).^1^ Orthologous genes in *A. thaliana* were used to perform the analysis (Chao et al., 2017; Raboanatahiry et al., 2017, 2018). The genes were clustered using Panther GO-slim biological process (Mi et al., 2021),^2^ and the interaction was visualized with Cytoscape_V3.6.0 (Shannon et al., 2003).

### Gene Expression and Structural Variation Analyses of Candidate Genes

The gene expression was obtained from BnTIR (Liu et al., 2021), because *ZS11* is the only rapeseed variety available in the BnTIR database, it was used as a reference for this analysis. *ZS11* genes were acquired from the BnPIR database (Song et al., 2021) and the genes expression analysis was obtained from the BnTIR database (Liu et al., 2021). The heatmap was built using Heatmapper (Babicki et al., 2016).^3^ The developing siliques and the seeds of *KenC-8* were collected 30 days after flowering for the q-PCR analysis, and *Actin7* was used as an internal control.

The identification of homologous candidate genes in *Gangan, No2127, Quinta, Shengli, Tapidor, Westar, Zheyou7*, and *ZS11* was made with a blast tool in the BnPIR database (Song et al., 2021),^4^ using *Darmor-bzh* gene sequences as a query sequence. *In silico* gene expression analysis was made using the “gene expression” tool of BnPIR. The identity percentage between CDS sequences was calculated using Vector NTI Advance 11.5.1.

## Data Availability Statement

The original contributions presented in the study are included in the article/Supplementary Material, further inquiries can be directed to the corresponding author/s.

## Author Contributions

NR wrote the manuscript. NR and HC performed the analysis of the QTL alignment map and candidate genes identification. NR and JH made the synteny analysis and the gene interaction network. NR, HL, and YY analyzed the gene expression. ML supervised the work and revised the manuscript. All authors contributed to the article and approved the submitted version.

## Conflict of Interest

The authors declare that the research was conducted in the absence of any commercial or financial relationships that could be construed as a potential conflict of interest.

## Publisher’s Note

All claims expressed in this article are solely those of the authors and do not necessarily represent those of their affiliated organizations, or those of the publisher, the editors and the reviewers. Any product that may be evaluated in this article, or claim that may be made by its manufacturer, is not guaranteed or endorsed by the publisher.

## Footnotes

1
http://string-db.org/

2
http://pantherdb.org/

3
http://www2.heatmapper.ca/

4
http://cbi.hzau.edu.cn/bnapus/
