# Supplementary material for: Construction of a Quantitative Genomic Map, Identification and Expression Analysis of Candidate Genes for Agronomic and Disease-Related Traits in Brassica napus
Source: Front Plant Sci. 2022 Mar 11;13:862363. doi: 10.3389/fpls.2022.862363 (PMC8963808; doi:10.3389/fpls.2022.862363)
Supplement: Supplementary file 14 [file Table_14.pdf]

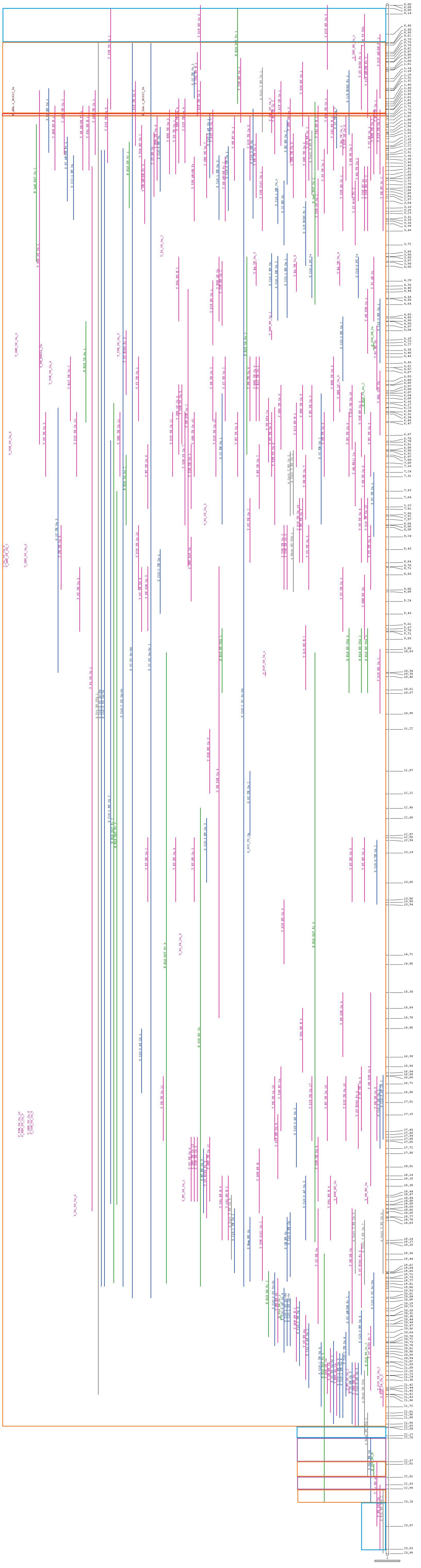

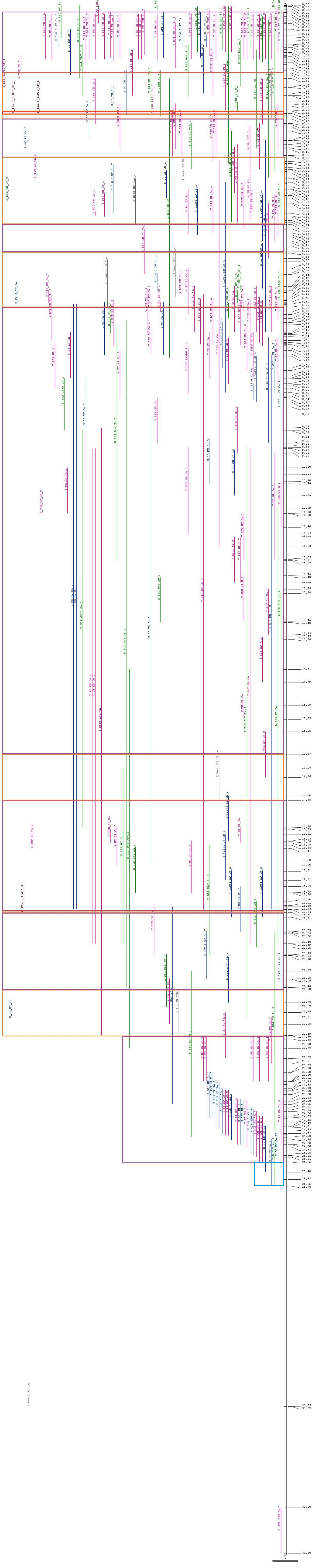

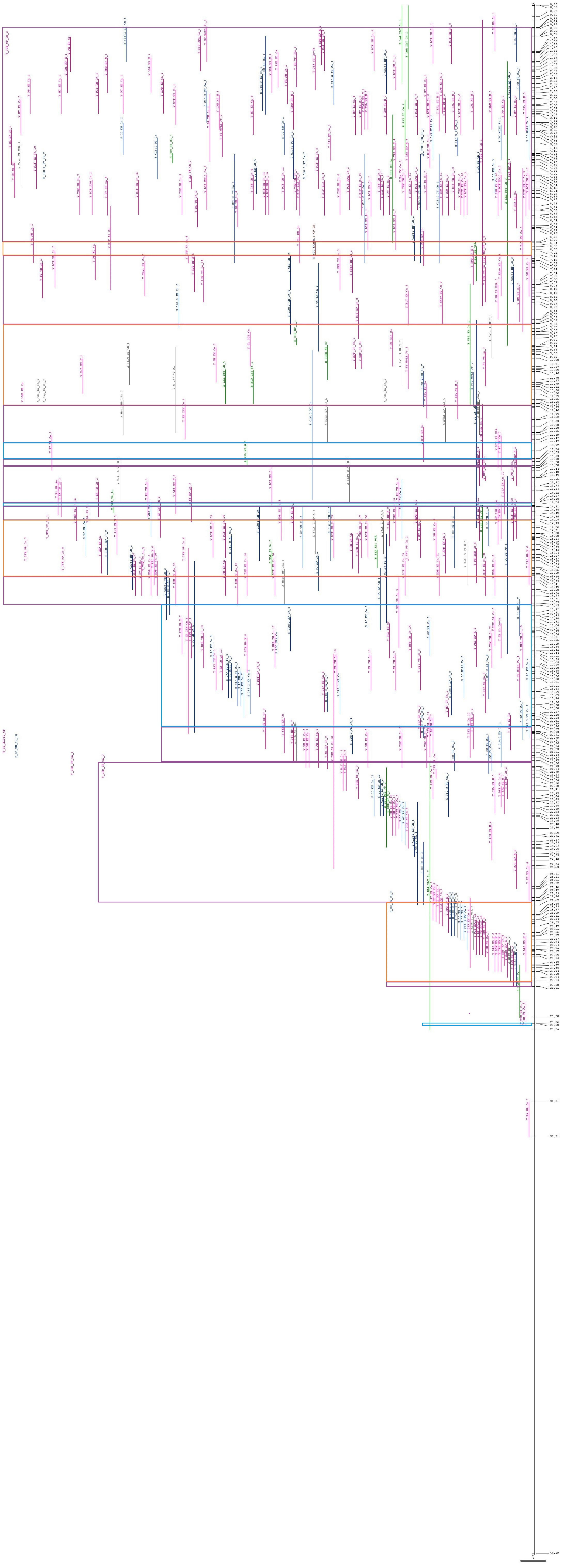

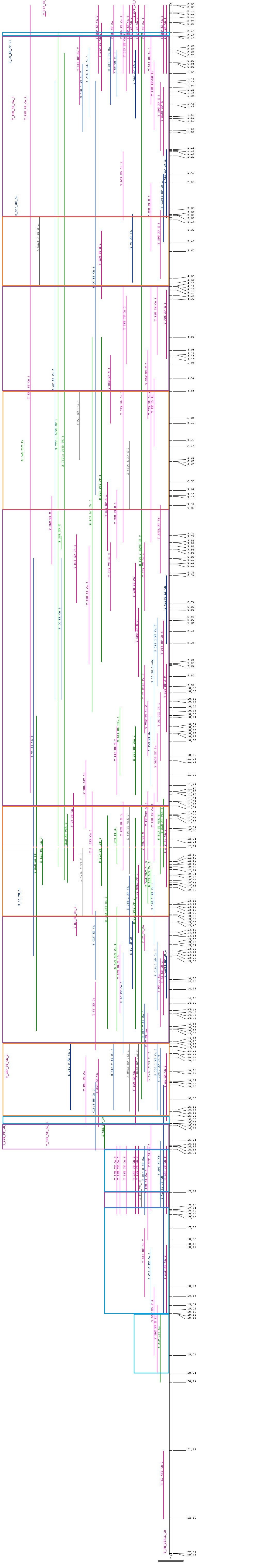

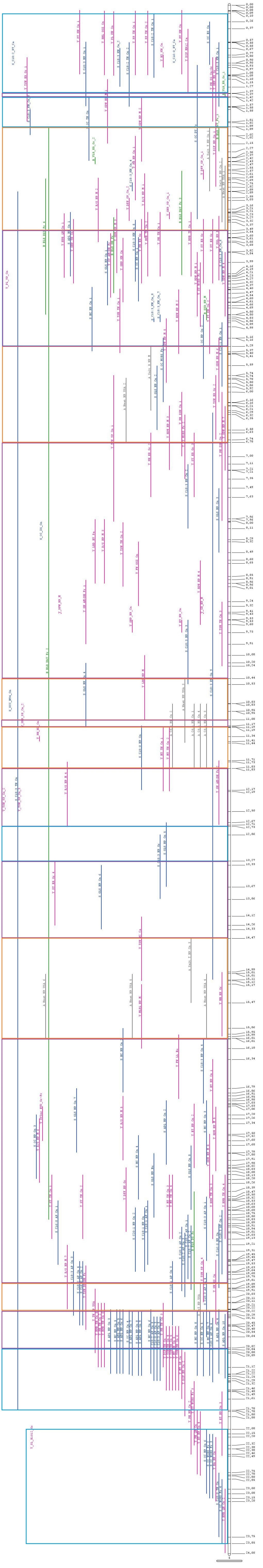

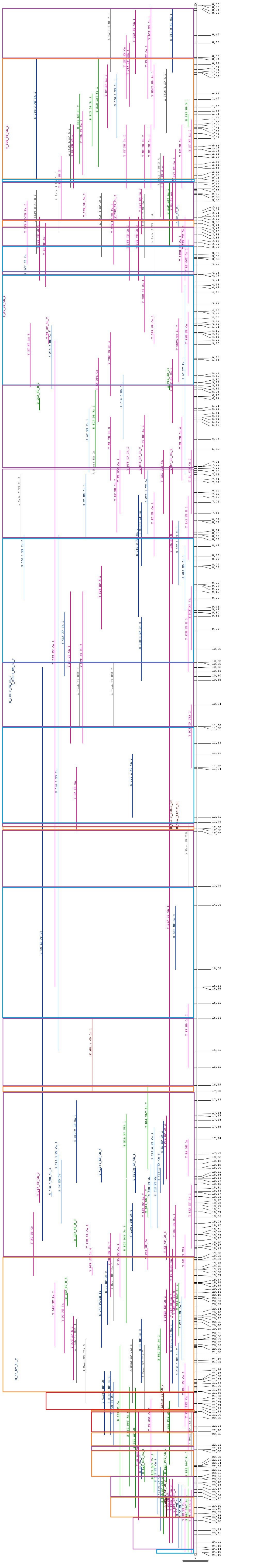

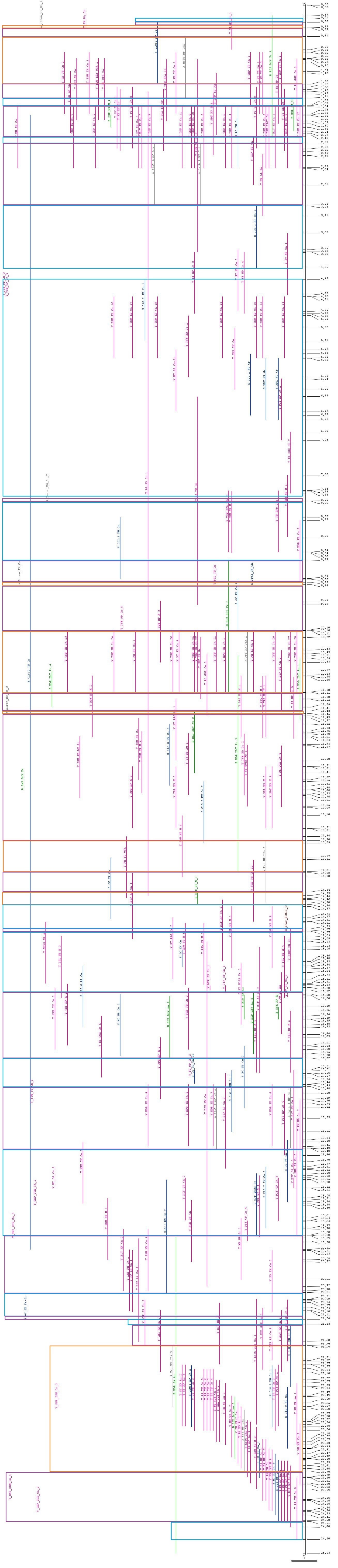

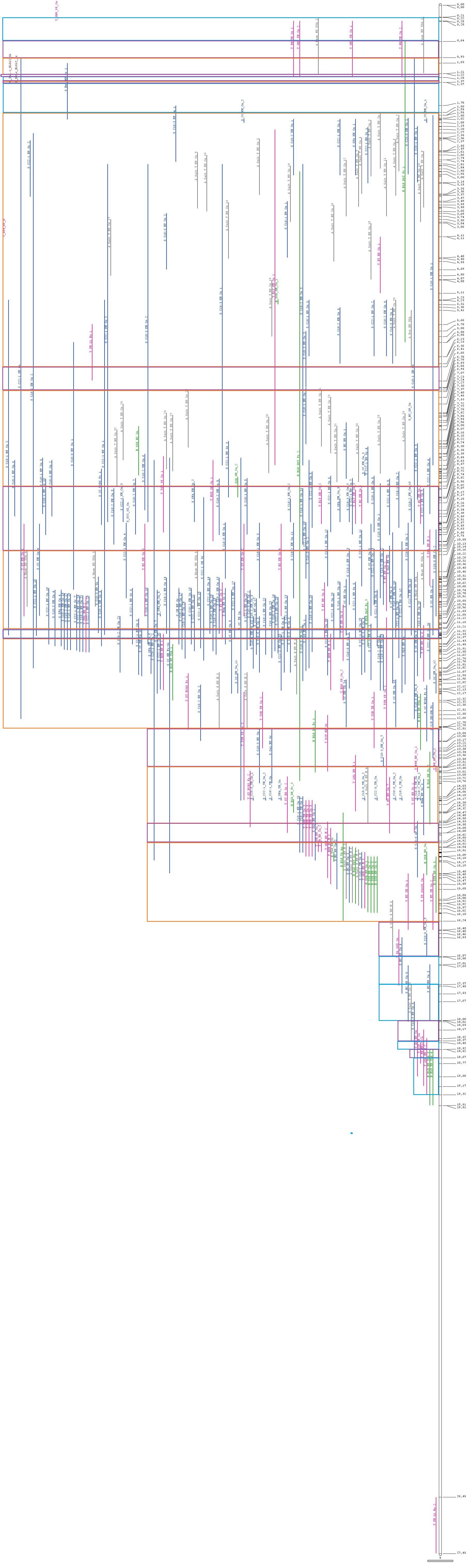

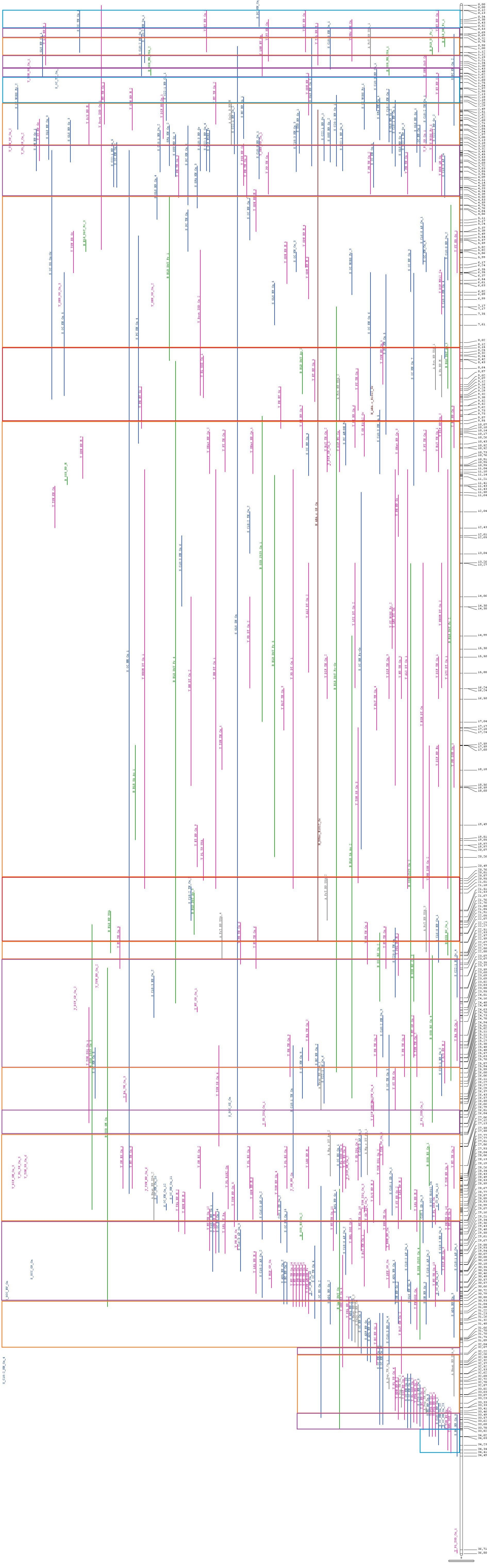

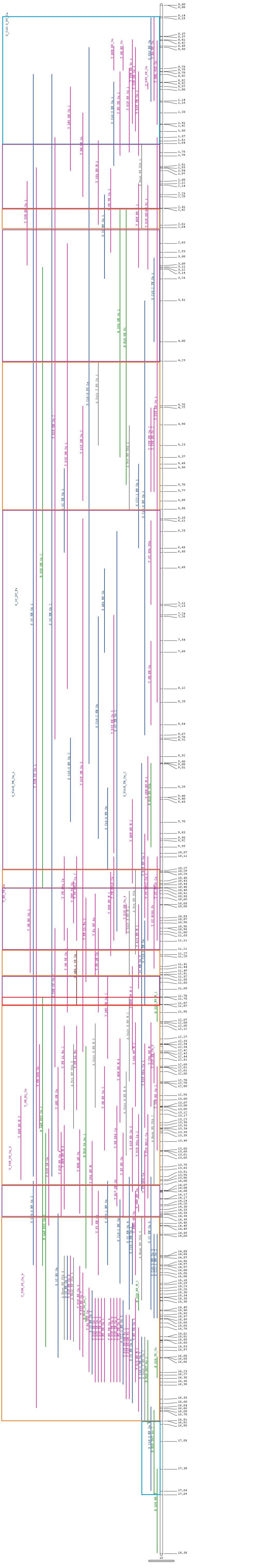

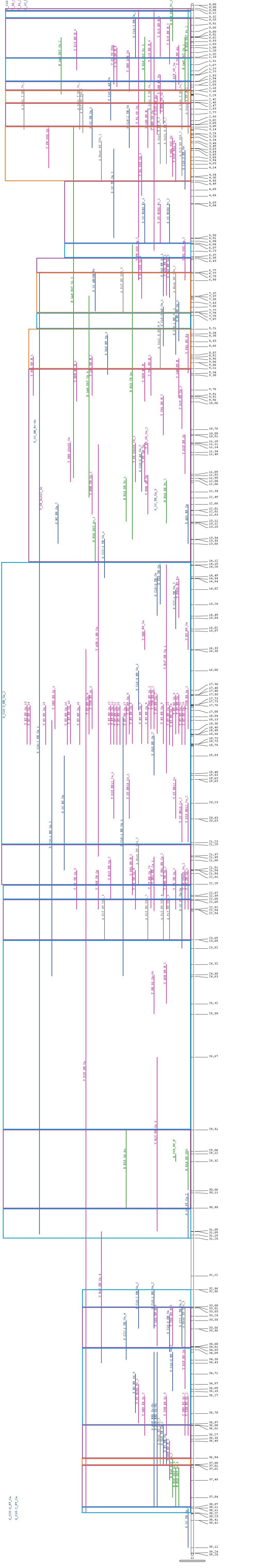

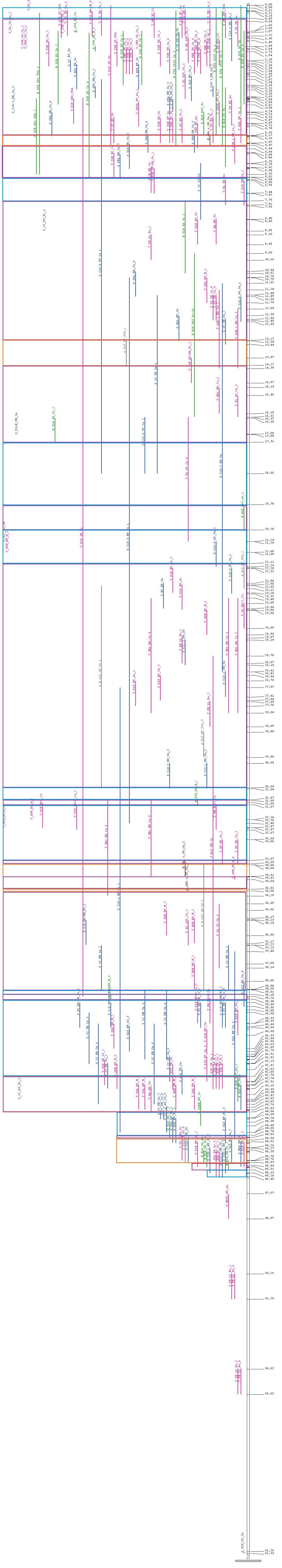

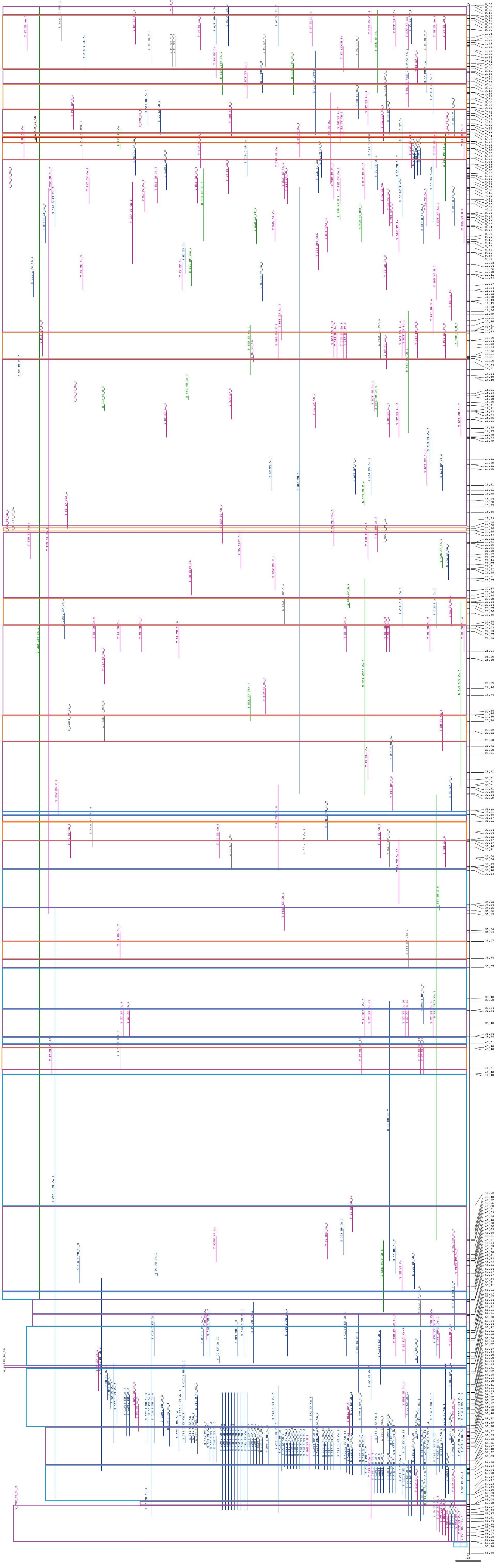

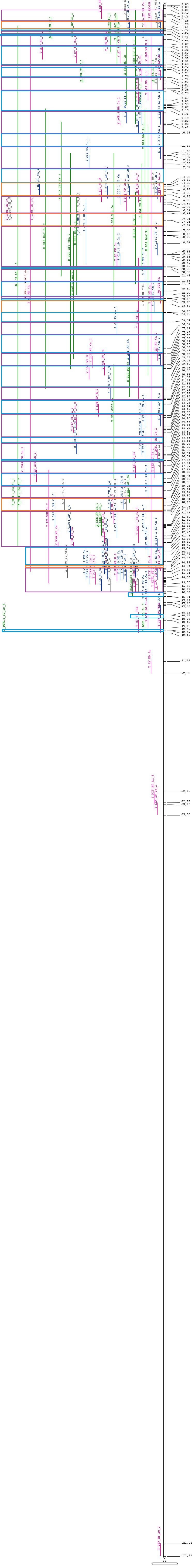

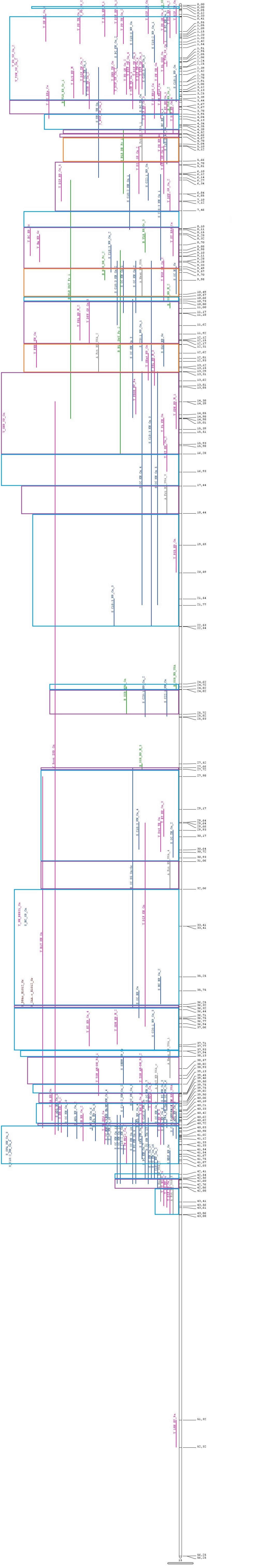

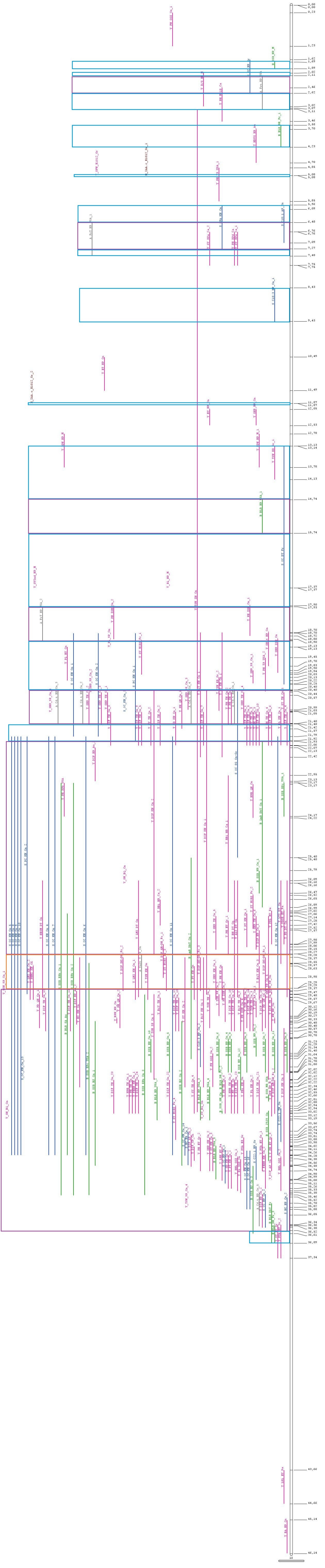

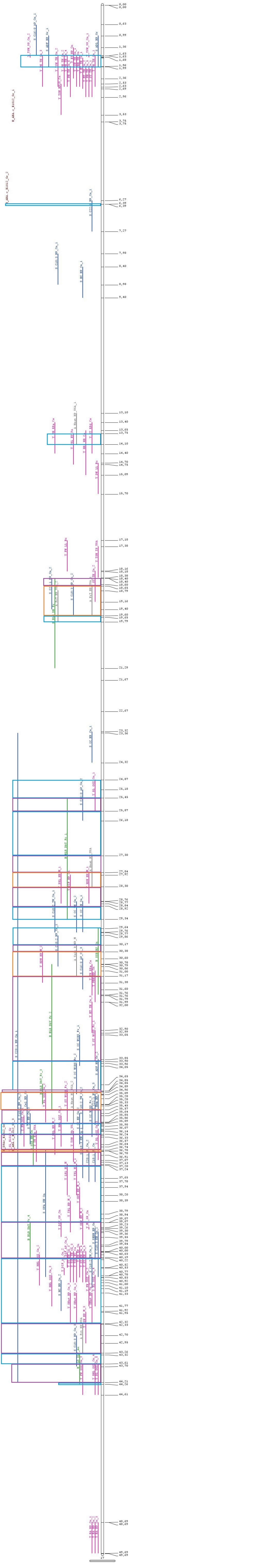

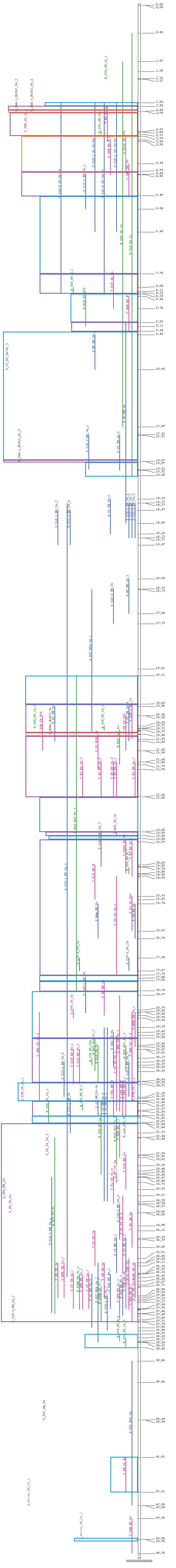

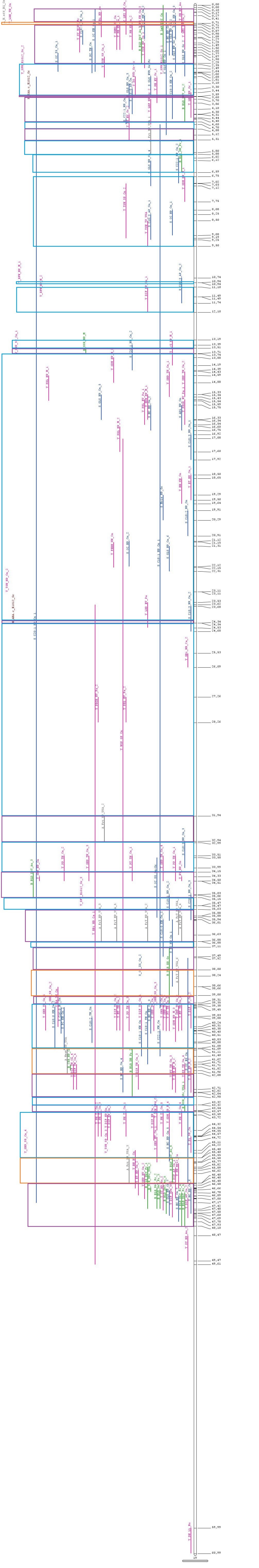

Supplementary figure 1. Alignment of QTLs on the physical map of *Darmor-bzh.*
The 4555 QTLs were aligned on the 19 chromosomes of *Darmor-bzh*. Different color corresponds to different categories of traits: gray (abiotic factor/disease), green (biotic factor/disease), garnet red (hormone level), blue (seed composition) and pink (yield related traits). The name category and name of trait is written on each QTL followed by the name of traits, the population and environment of origin. Location on the physical map is on the right side of the map. The overlapping QTLs are highlighted inside squares, according to the number of categories involved: red (5 categories), orange (4 categories), purple (3 categories), blue (2 categories). The names of chromosome are in the bottom (A1-A10 = 1-10; C1-C9 = 11-19). The map was built using BioMercator V4.2 software (Sosnowski and Joets, 2012).
