# Supplementary material for: Construction of a Quantitative Genomic Map, Identification and Expression Analysis of Candidate Genes for Agronomic and Disease-Related Traits in Brassica napus
Source: Front Plant Sci. 2022 Mar 11;13:862363. doi: 10.3389/fpls.2022.862363 (PMC8963808; doi:10.3389/fpls.2022.862363)
Supplement: Supplementary file 15 [file Table_15.pdf]

FUS3

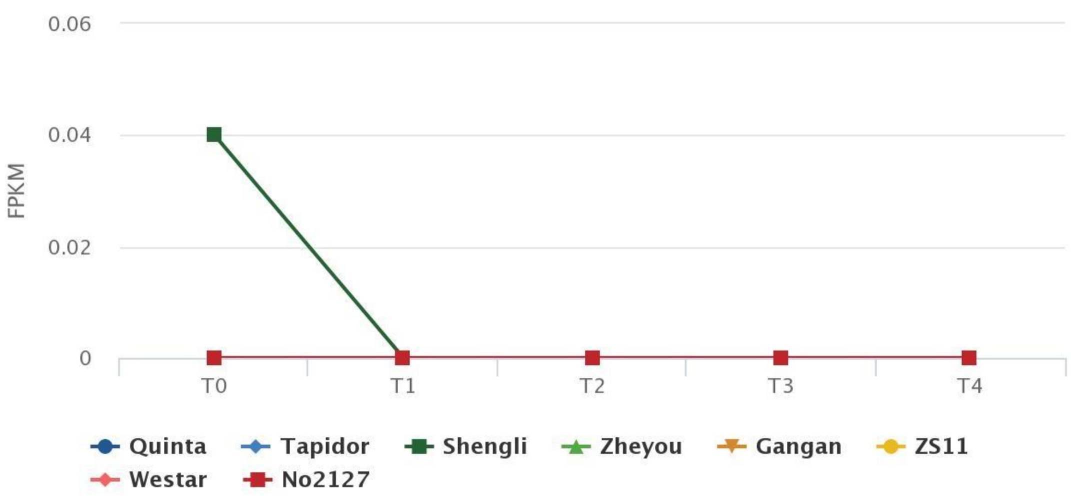

PRP4 KINASE B

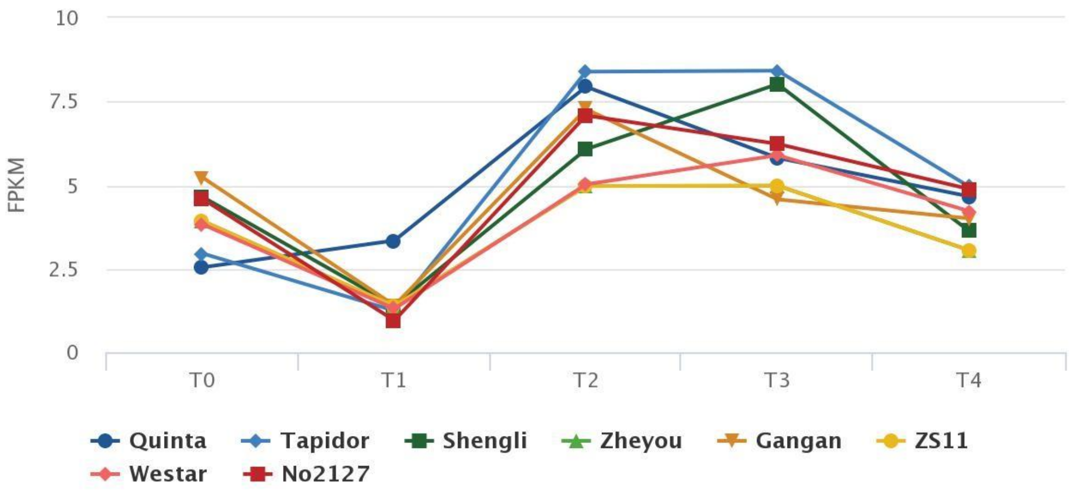

KR

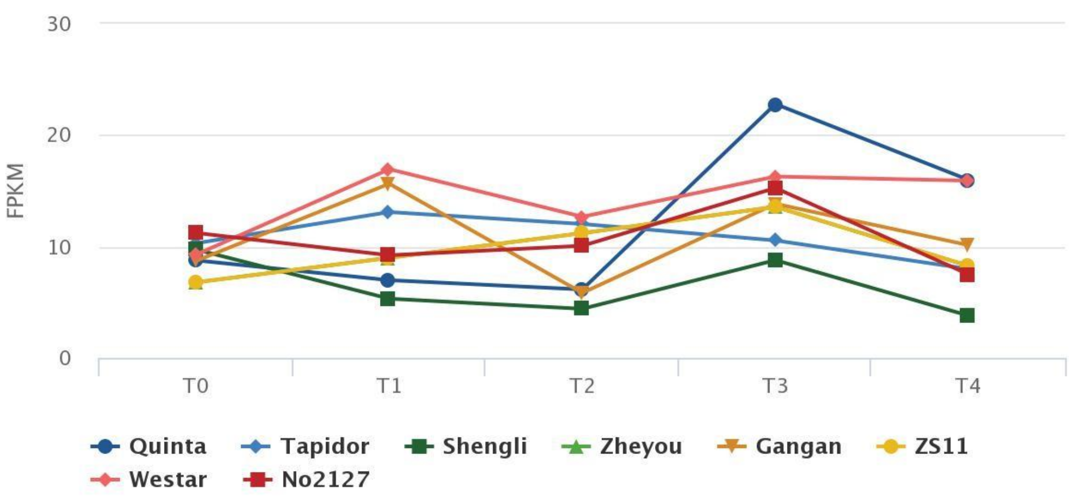
30
20
10
0
FPKM
T0
T1
T2
T3
T4
Quinta
Tapidor
Shengli
Zheyou
Gangan
ZS11
Westar
No2127

RCN1

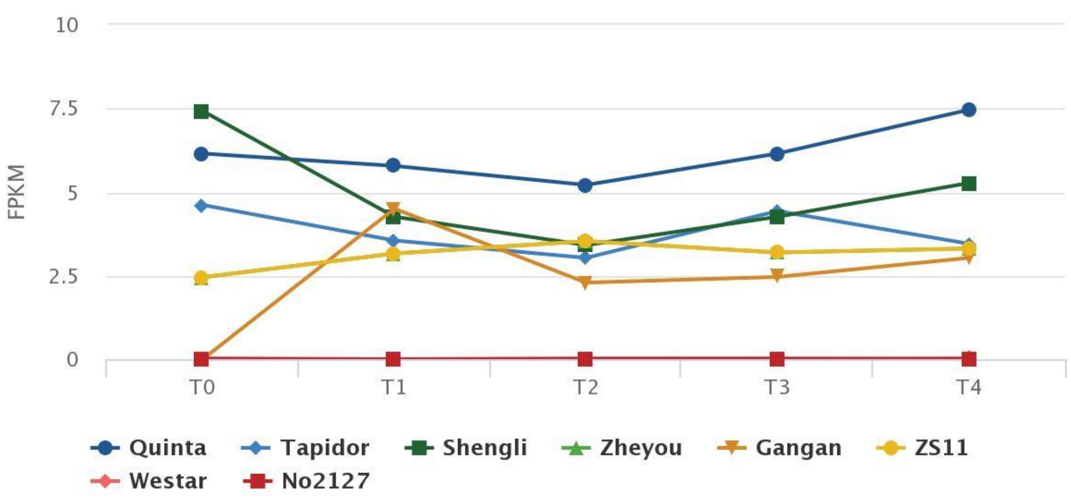
10
7.5
5
2.5
0
FPKM
T0
T1
T2
T3
T4
Quinta
Tapidor
Shengli
Zheyou
Gangan
ZS11
Westar
No2127

KAS-I

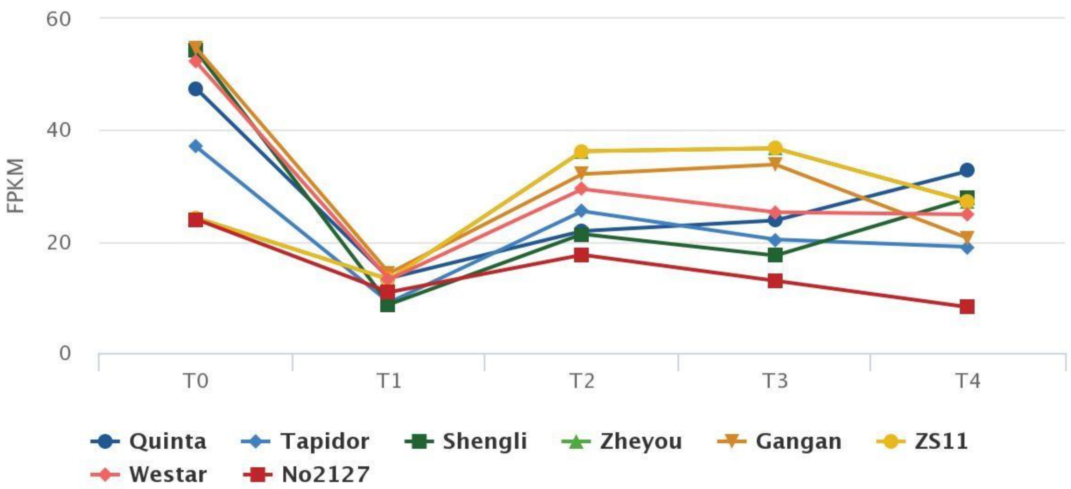
60
40
20
0
FPKM
T0
T1
T2
T3
T4
Quinta
Tapidor
Shengli
Zheyou
Gangan
ZS11
Westar
No2127

KAS-III

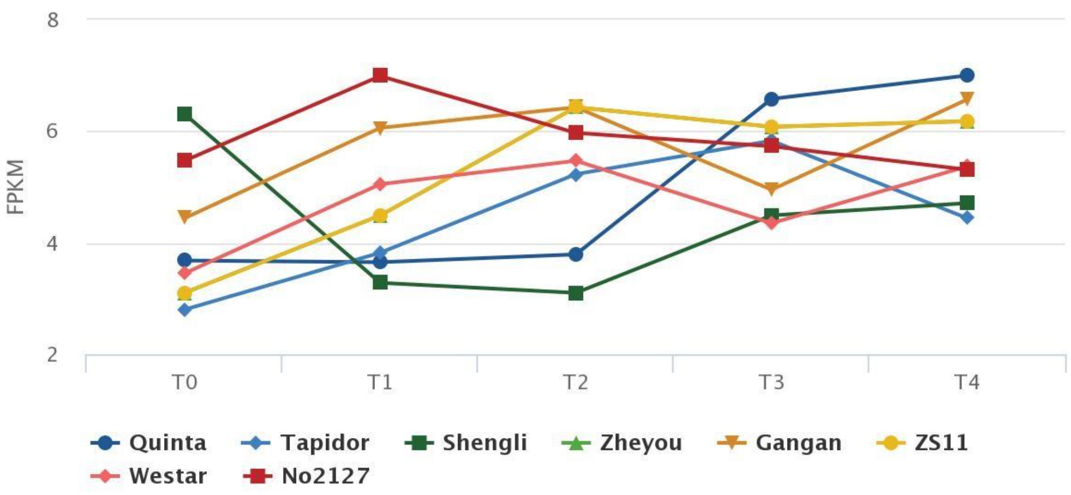
8
6
4
2
FPKM
T0
T1
T2
T3
T4
Quinta
Tapidor
Shengli
Zheyou
Gangan
ZS11
Westar
No2127

MCAT

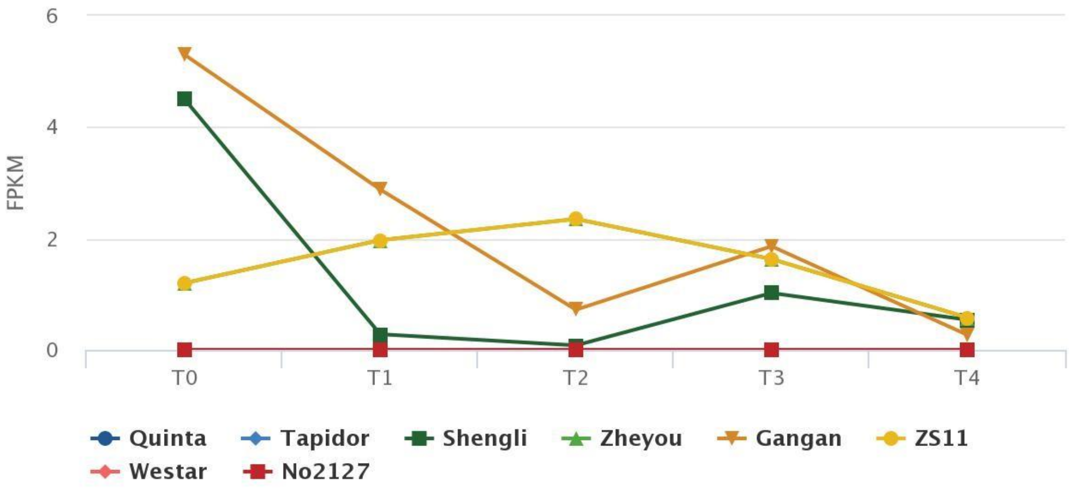

FPKM
6
4
2
0
T0
T1
T2
T3
T4
Quinta
Tapidor
Shengli
Zheyou
Gangan
ZS11
Westar
No2127

FLC

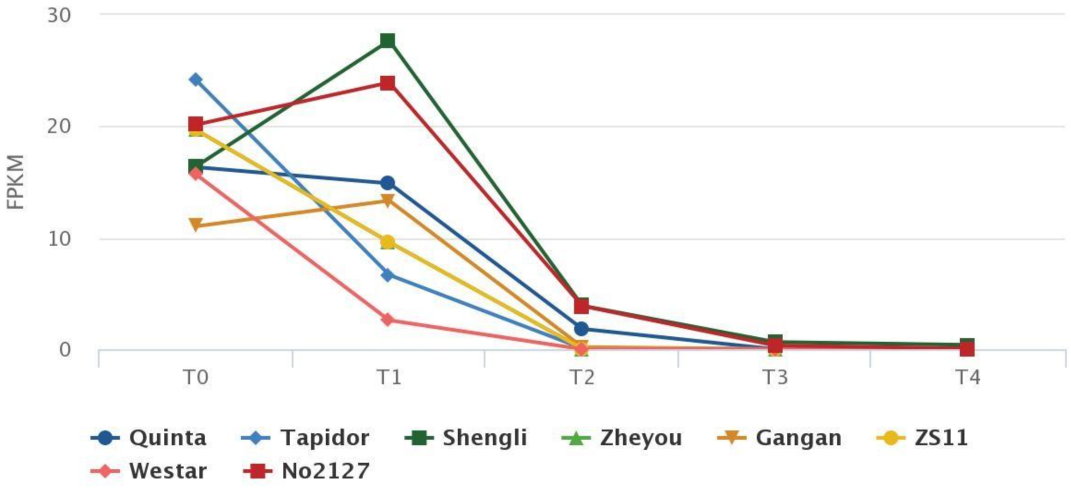

FPKM
30
20
10
0
T0
T1
T2
T3
T4
Quinta
Tapidor
Shengli
Zheyou
Gangan
ZS11
Westar
No2127

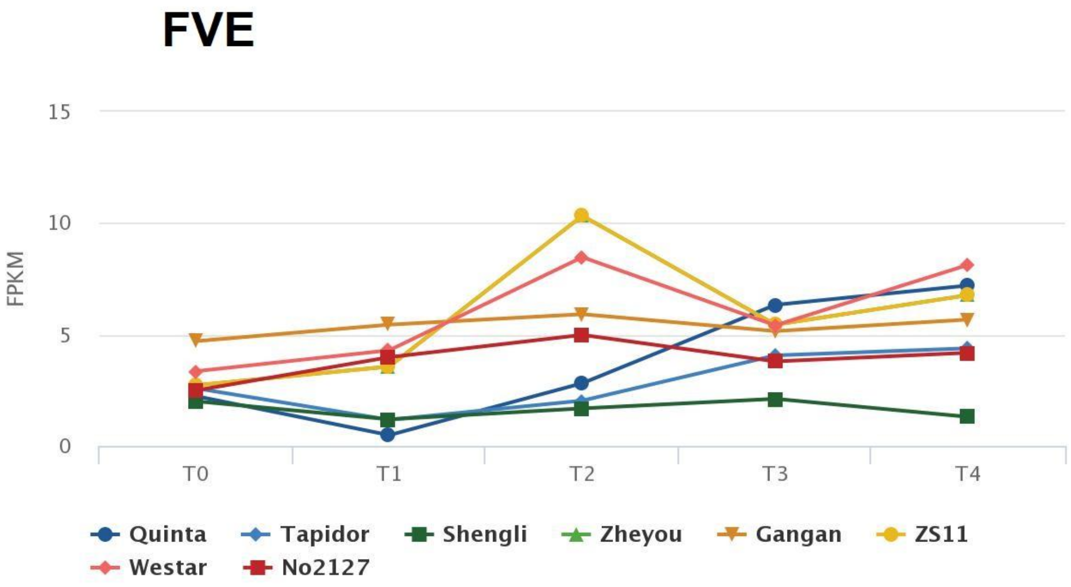

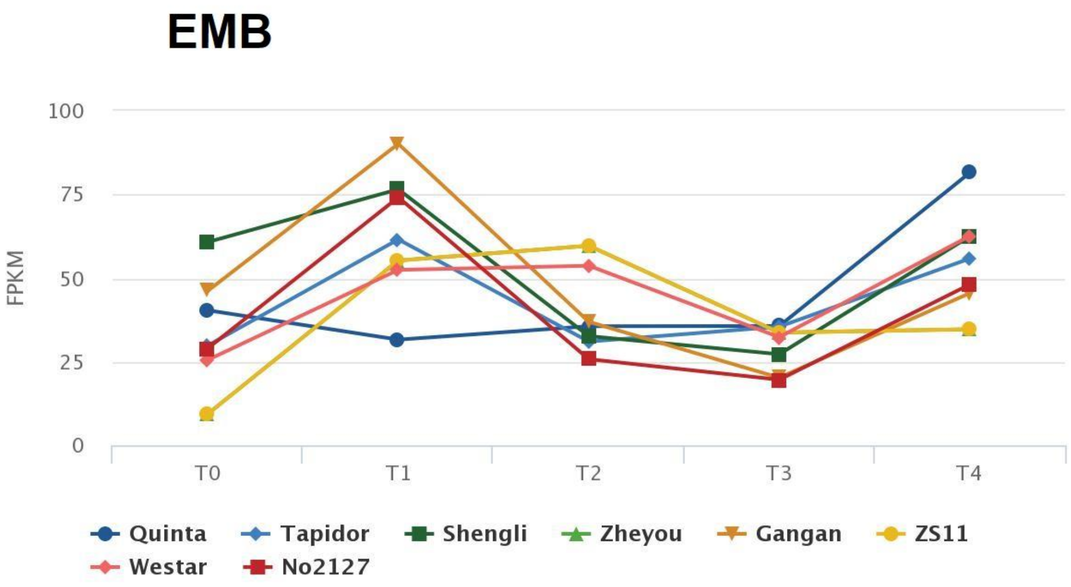

Supplementary figure 2. Gene expression of genes in eight varieties of rapeseed. The tissues used in the determination of gene expression on the BnPIR database were collected throughout the flowering process of the plant from the eight accessions of this study. They were collected at five different post sowing days: T0: 24 days post sowing; T1: 54 days post sowing; T2: 82 days post sowing; T3: 115 days post sowing; T4: 147 days post sowing.
